# Supplementary material for: Carbon nanotubes as excitonic insulators
Source: Nat Commun. 2017 Nov 13;8:1461. doi: 10.1038/s41467-017-01660-8 (PMC5684388; doi:10.1038/s41467-017-01660-8)
Supplement: Supplementary file 1 — Supplementary Information [file 41467_2017_1660_MOESM1_ESM.pdf]

Supplementary Note 1

Effective-mass theory of armchair carbon nanotubes

In this Note we recall the effective-mass theory of electronic π-states in single-wall carbon nanotubes, focusing on the lowest conduction and highest valence band of undoped armchair tubes[1–3]. Carbon nanotubes may be thought of as wrapped sheets of graphene, hence nanotube electronic states are built from those of graphene through a folding procedure, after quantizing the transverse wave vector. Low-energy graphene states belong to one of the two Dirac cones, whose apexes intersect the degenerate K and K′ points, respectively, at the corners of graphene first Brillouin zone. At these two points the energy gap is zero.

Close to Brillouin zone corners $\tau = \mathrm{K}, \mathrm{K}'$, a nanotube state $\psi(\mathbf{r})$ is the superposition of slowly-varying envelope functions $F^{\tau\eta}(\mathbf{r})$ multiplied by the Bloch states $\psi_{\tau\eta}(\mathbf{r})$, the latter having two separate components localized on sublattices $\eta = A$ and $\eta = B$, respectively (cyan and red dots in Supplementary Fig. 1):

$$\psi(\mathbf{r}) = \sum_{\tau=\mathrm{K},\mathrm{K}'} \sum_{\eta=A,B} F^{\tau\eta}(\mathbf{r})\, \psi_{\tau\eta}(\mathbf{r}). \tag{1}$$

The effective-mass approximation of Supplementary Eq. (1) goes beyond the usual one-valley treatment, as below we explicitly consider intervalley coupling due to Coulomb interaction. The relative phases of different Bloch state components $\psi_{\tau\eta}$ are fixed by symmetry considerations, as

detailed in Supplementary Note 7. The envelope $F^{\tau\eta}$ is a pseudospinor with respect to valley and sublattice indices, $\boldsymbol{F} \equiv (F^{\mathrm{K}A}, F^{\mathrm{K}B}, F^{\mathrm{K}'A}, F^{\mathrm{K}'B})^T$. In the valley-sublattice product space, $\boldsymbol{F}$ obeys the Dirac equation of graphene:

$$\gamma \left[\boldsymbol{\sigma}_x \otimes \mathbf{1}_\tau \hat{k}_x + \boldsymbol{\sigma}_y \otimes \boldsymbol{\tau}_z \hat{k}_y \right] \boldsymbol{F}(\mathbf{r}) = \varepsilon\, \boldsymbol{F}(\mathbf{r}). \tag{2}$$

Here $\boldsymbol{\sigma}_x$ and $\boldsymbol{\sigma}_y$ are 2×2 Pauli matrices acting on the sublattice pseudospin, $\boldsymbol{\tau}_z$ and the 2×2 identity matrix $\mathbf{1}_\tau$ act on the valley pseudospin, $\hat{k}_x = -i\partial/\partial x$ is is the wave vector operator along the circumference direction x and $\hat{k}_y = -i\partial/\partial y$ acts on the tube axis coordinate y, γ is graphene's band parameter, and ε is the single-particle energy. Furthermore, $\boldsymbol{F}$ obeys the boundary condition along the tube circumference:

$$\boldsymbol{F}(\mathbf{r} + \mathbf{L}) = \boldsymbol{F}(\mathbf{r}) \exp\left(2\pi i\, \varphi\right), \tag{3}$$

where $\mathbf{L}$ is the chiral vector in the circumference direction of the tube and $|\mathbf{L}| = L = 2\pi R$ is the circumference. A magnetic field may or may not be applied along the tube axis, with $\varphi = \phi/\phi_0$ being the ratio of the magnetic flux ϕ piercing the tube cross section to the magnetic flux quantum $\phi_0 = ch/e$. Supplementary Eq. (2) depends on the reference frame. Note that in our effective-mass treatment the x and y directions are parallel to the circumference and axis of the tube, respectively, as shown in Supplementary Fig. 1a, whereas in the main text as well as in the first-principles treatment the z axis is parallel to the tube.

The energy bands are specified by the valley index τ, the valence index $\alpha = c,\, v$ denoting either the conduction ($\alpha = c$) or the valence band ($\alpha = v$), and the wave vector k in the axis direction. The wave functions in K and K′ valleys are respectively $\boldsymbol{F} \equiv (\boldsymbol{F}^{\mathrm{K}}_{\alpha k}(\mathbf{r}), 0)^T$ and $(0, \boldsymbol{F}^{\mathrm{K}'}_{\alpha k}(\mathbf{r}))^T$,

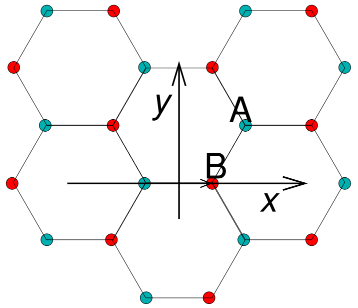

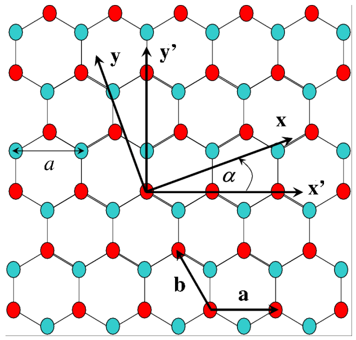

Supplementary Fig. 1 Carbon nanotube reference frames for the effective-mass model. **a** Reference frame for the armchair tube used in this work. The x and y directions are parallel to the circumference and axis of the tube, respectively. The small vector is $\mathbf{R}_0^B$, i.e., the basis vector locating the origin of the B sublattice. Cyan and red dots point to A and B sublattices, respectively. **b** Ando's reference frame for a generic tube. The frame origin is located on an atom of the B sublattice. The tube frame is obtained by rotating the $x'y'$ graphene reference frame by the chiral angle α. The chiral vector $\mathbf{L}$ identifying the tube circumference is $\mathbf{L} = -m\mathbf{a} - (n+m)\mathbf{b}$ in terms of the conventional chiral indices (n, m), where $\mathbf{a}$ and $\mathbf{b}$ are the primitive translation vectors of graphene shown in the picture. For an equivalent choice of $\mathbf{L}$ one has $\alpha = \pi/6$ for (n, n) armchair tubes and $\alpha = 0$ and for $(n, 0)$ zigzag tubes. a is the lattice constant of graphene.

with $\boldsymbol{F}^{\tau}_{\alpha k}(\mathbf{r}) \equiv (F^{\tau A}_{\alpha k}, F^{\tau B}_{\alpha k})^T$ being a plane-wave pseudospinor in the sublattice space,

$$\boldsymbol{F}^{\tau}_{\alpha k}(\mathbf{r}) = \boldsymbol{\xi}^{\tau}_{\alpha k}(x) \frac{1}{\sqrt{A}} \exp{(iky)}, \tag{4}$$

where A is the tube length and the wave function $\boldsymbol{\xi}^{\tau}_{\alpha k}(x)$ for the motion along the circumference direction is

$$\boldsymbol{\xi}^{\tau}_{\alpha k}(x) = \frac{1}{\sqrt{L}} \exp{(ik_{\perp} x)} \boldsymbol{F}_{\tau \alpha k}. \tag{5}$$

The constant pseudospinor $\boldsymbol{F}_{\tau \alpha k}$ is a unit vector with a k-dependent phase between the two sublattice components,

$$\boldsymbol{F}_{\mathrm{K}\alpha k} = \frac{1}{\sqrt{2}} \begin{pmatrix} b(k) \\ s_{\alpha} \end{pmatrix}, \qquad \boldsymbol{F}_{\mathrm{K}'\alpha k} = \frac{1}{\sqrt{2}} \begin{pmatrix} b^{*}(k) \\ s_{\alpha} \end{pmatrix}, \tag{6}$$

where

$$b(k) = \frac{k_{\perp} - ik}{\sqrt{k_{\perp}^2 + k^2}}, \tag{7}$$

and $s_{\alpha} = \pm 1$ for conduction and valence bands, respectively. In Supplementary Eqs. (5) and (7) the transverse wave vector $k_{\perp}$ is proportional to the magnetic flux φ,

$$k_{\perp} = \frac{\varphi}{R}. \tag{8}$$

In each valley, the energy is

$$\varepsilon_{\alpha}(k) = s_{\alpha} \gamma \sqrt{k_{\perp}^2 + k^2}, \tag{9}$$

where the origin of the k axis is located at the Dirac point K (K$'$).

Figure 2a of main text shows the first-principles band structure of the (3,3) tube in a range of a few eV around the Dirac point, with k scanning half Brillouin zone, between the origin ($k = 0$, Γ point) and $k = \pi/a$ ($a = 2.46$ Å is graphene lattice constant). The negative k axis, containing the

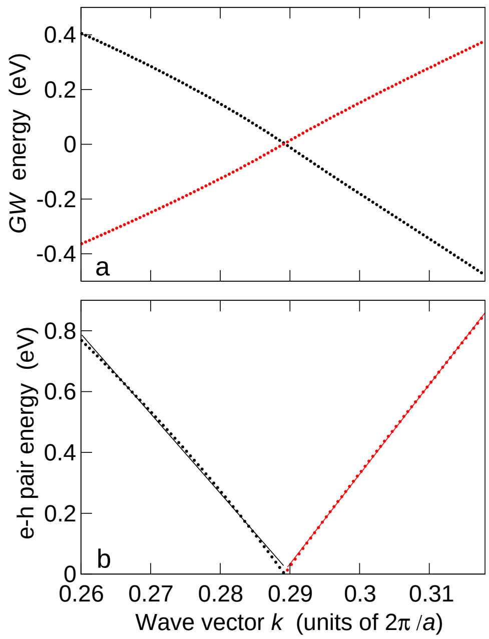

Supplementary Fig. 2 GW band structure of the (3,3) tube. **a** GW band structure vs wave vector k close to the Dirac point K. Red [grey] and black dots point to chirality indices $\mathcal{C} = +1$ and -1, respectively. **b** Electron-hole pair excitation energy vs k. The lines are linear fits to the data.

K′ point, is obtained by specular reflection. The DFT / GW location of the Dirac point is K = 0.289 $(2\pi/a)$, whereas the effective-mass estimate is K = $1/3$ $(2\pi/a)$ (the discrepancy between DFT and tight-binding predictions is well documented in the literature[4,5]). As seen in Supplementary Fig. 2a, the GW bands are approximately linear in an energy range of at least ± 0.4 eV around the Dirac point, which validates the effective-mass model at low energy.

Note that, in the absence of the magnetic field, electron states have a well defined chirality[6–8], which is one of the two projections, $\mathcal{C}$, of the sublattice pseudospin onto the momentum direction, expressed as the eigenvalues $\mathcal{C} = \pm 1$ of the operator $\boldsymbol{\sigma}_y \otimes \boldsymbol{\tau}_z$. The chirality index is highlighted by red ($\mathcal{C} = +1$) and black ($\mathcal{C} = -1$) colour in Supplementary Fig. 2a.

Supplementary Note 2

Electron-electron interaction: Effective-mass vs first-principles description

Within the effective-mass framework, the Coulomb interaction v between two electrons on the carbon nanotube cylindrical surface located at $\mathbf{r} \equiv (x, y)$ and $\mathbf{r}' \equiv (x', y')$, respectively, is[2]

$$v(\mathbf{r}, \mathbf{r}') = \sum_q e^{iq(y-y')} \frac{2e^2}{\kappa_{\mathrm{r}} A} K_0\left(2R \left| q \sin\left(\frac{x - x'}{2R}\right)\right|\right), \tag{10}$$

where κ_{r} is a static dielectric constant that takes into account polarization effects due to the electrons not included in the effective-mass description plus the contribution of the dielectric back-

ground. The interaction matrix element between single-particle states is[9,10]

$$
\begin{aligned}
& V_{(\tau,\alpha,k+q),(\tau',\beta',k');(\tau',\alpha',k'+q)(\tau,\beta,k)} \\
= & \int\! d\mathbf{r} \int\! d\mathbf{r}' \; [\boldsymbol{F}^{\tau}_{\alpha k+q}(\mathbf{r})]^{\dagger} \cdot \boldsymbol{F}^{\tau}_{\beta k}(\mathbf{r}) \; v(\mathbf{r},\mathbf{r}') \; [\boldsymbol{F}^{\tau'}_{\beta' k'}(\mathbf{r}')]^{\dagger} \cdot \boldsymbol{F}^{\tau'}_{\alpha' k'+q}(\mathbf{r}') \\
= & \frac{1}{A} \boldsymbol{F}^{\dagger}_{\tau\alpha k+q} \cdot \boldsymbol{F}_{\tau\beta k} \;\; \boldsymbol{F}^{\dagger}_{\tau'\beta' k'} \cdot \boldsymbol{F}_{\tau'\alpha' k'+q} \; v(q),
\end{aligned}
\tag{11}
$$

where the one-dimensional effective interaction resolved in momentum space,

$$
v(q) = \frac{2e^2}{\kappa_{\rm r}} \, I_0(R\,|q|) \; K_0(R\,|q|)\,, \tag{12}
$$

is modulated by a form factor given by overlap terms between sublattice pseudospinors [$I_0(z)$ and $K_0(z)$ are the modified Bessel functions of the first and second kind, respectively[11]]. The effect of screening due to the polarization of those electrons that are treated within the effective-mass approximation is considered by replacing $v(q)$ with

$$
w(q) = \frac{v(q)}{\varepsilon(q)} \tag{13}
$$

in the matrix element (11), where $\varepsilon(q)$ is the static dielectric function (to discriminate between screened and unscreened matrix elements we use respectively ‘w’ and ‘v’ letters throughout the Supplementary Information). It may be shown that dynamical polarization effects are negligible in the relevant range of small frequencies, which is comparable to exciton binding energies.

Note that terms, similar to Supplementary Eq. (11), that scatter electrons from one valley to the other are absent in the effective mass approximation. These small intervalley terms, as well as the interband exchange terms, which are both induced by the residual, short-range part of Coulomb interaction, are discussed in Supplementary Note 3.

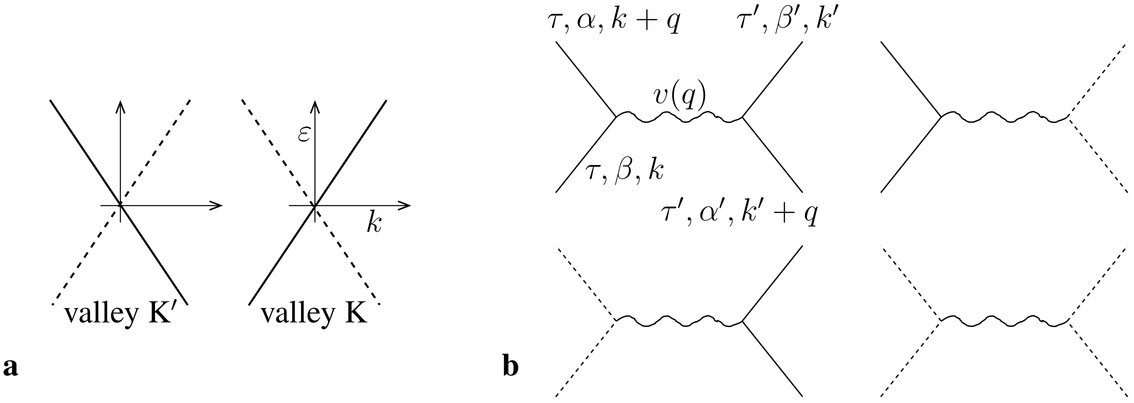

Supplementary Fig. 3 Effect of chirality on Coulomb interaction matrix elements. **a** Energy bands and chiralities of electron states in armchair carbon nanotubes in the absence of the magnetic field. Solid and dashed lines highlight chirality $\mathcal{C} = \pm 1$, respectively. **b** Allowed scattering processes induced by long-range Coulomb interaction. The indices $\tau = \mathrm{K}, \mathrm{K}'$ and $\alpha = c, v$ label valleys and bands, respectively. The chirality is conserved at each vertex of diagrams.

Effect of chiral symmetry. The chiralities of electron states, which is illustrated in Supplementary Fig. 3a (solid and dashed lines label $\mathcal{C} = +1$ and $\mathcal{C} = -1$, respectively), signficantly affects Coulomb interaction matrix elements. This occurs through the form factors of the type $\boldsymbol{F}^{\dagger}\cdot\boldsymbol{F}$ appearing in Supplementary Eq. (11), which are overlap terms between sublattice pseudospinors. As apparent from their analytical structure,

$$\boldsymbol{F}^{\dagger}_{\tau\alpha k+q} \cdot \boldsymbol{F}_{\tau\beta k} = \frac{1}{2}\left[\,\mathrm{sign}(k)\,\mathrm{sign}(k+q) + s_{\beta}s_{\alpha}\right], \tag{14}$$

the chiral symmetry of the states is conserved at each vertex of Coulomb scattering diagrams (see Supplementary Fig. 3b), hence initial and final states scattered within the same band must have the same momentum direction. This significantly affects the Bethe-Salpeter equation for

excitons, as we show below. We are especially interested in the dominant long-range Coulomb matrix element[12] that binds electrons and holes:

$$V_{(\tau,c,k+q),(\tau,v,k);(\tau,v,k+q)(\tau,c,k)} \equiv \frac{\tilde{V}(k+q,k)}{A}. \tag{15}$$

This term scatters electron-hole pairs from the initial pair state $(c,k)(v,k)$ to the final state $(c,k+q)(v,k+q)$ within the same valley τ. Throughout this Supplementary Information we use the tilde symbol for quantities whose dimension is an energy multiplied by a length, like $V = \tilde{V}/A$.

In the first instance we neglect screening, since for low momentum transfer, $q \to 0$, polarization is suppressed hence $\varepsilon(q) \to 1$. In this limit Coulomb interaction diverges logarithmically,

$$v(q) \to -\frac{2e^2}{\kappa_{\mathrm{r}}} \ln(R\,|q|), \tag{16}$$

but this is harmless to the Bethe-Salpeter equation, since $v(q)$ occurs only in the kernel of the scattering term, hence it is integrated over q for macroscopic lengths A,

$$-\frac{1}{A}\sum_q \tilde{V}(k+q,k)\ldots \to -\frac{1}{2\pi}\int dq\,\tilde{V}(k+q,k)\ldots, \tag{17}$$

which removes the divergence. Note that, throughout this Supplementary Information and opposite to the convention of Fig. 2c of main text, we take V as a positive quantity. In detail, we discretize the momentum space axis, $k \to k_i$, where $k_i = i2\pi/(Na)$, $i = -N/2+1,\ldots,0,1,\ldots,N/2$, $N = A/a$ is the number of unitary cells, and $\Delta k = 2\pi/(Na)$ is the mesh used in the calculation. Hence, the regularized matrix element, integrated over the mesh, is

$$V(k_j+q_i,k_j) = \frac{1}{2\pi}\int_{q_i-\Delta k}^{q_i} dq\,\tilde{V}(k_j+q,k_j). \tag{18}$$

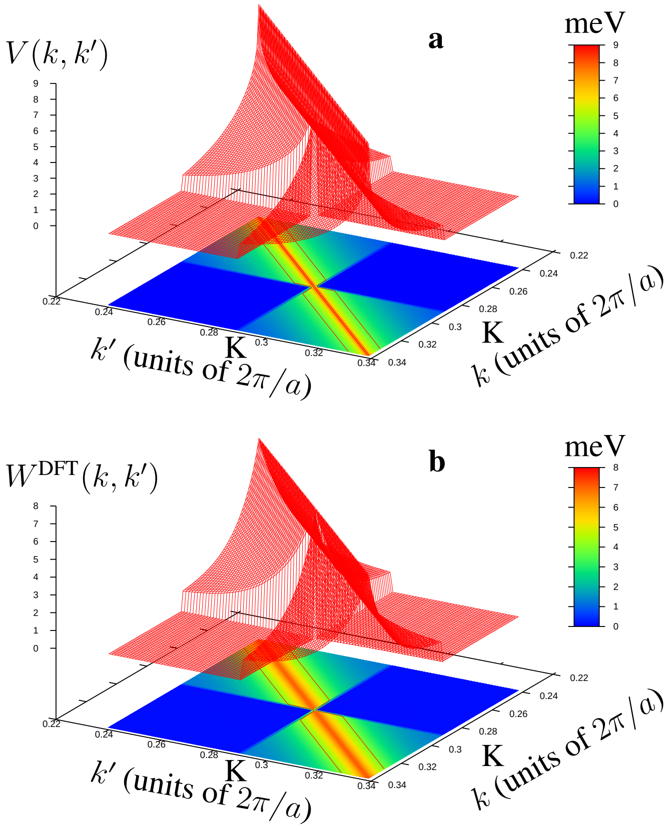

Supplementary Fig. 4 Dominant interband Coulomb matrix element in the (k, k') space close to the K point. **a** Effective-mass 'bare' matrix element $V(k, k')$, with $\kappa_{\rm r} = 10$ and $\varepsilon(k - k') = 1$. The isolines of the two-dimensional contour map point to the heights of 4 and 8 meV, respectively. **b** Modulus of DFT screened matrix element $W^{\rm DFT}(k, k')$ obtained within the random phase approximation for the $(3, 3)$ armchair tube. Here $N = 900$ and K = $0.289(2\pi)/a$.

In Supplementary Fig. 4 we compare V (panel a, $\kappa_{\rm r} = 10$) with the modulus of the screened DFT matrix element $W^{\rm DFT}$ obtained for the $(3, 3)$ tube (panel b). The two plots are three-dimensional contour maps in a square domain (k, k') centered around the Dirac point, with K = $0.289(2\pi)/a$ and $N = 900$. The two matrix elements agree almost quantitatively, as they both exhibit: (i) zero or very small values in the second and fourth quadrants, i.e., $k >$ K and $k' <$ K or $k <$ K and $k' >$ K (ii) a logarithmic spike on the domain diagonal, i.e., $k' \to k$. This behavior has a simple interpretation in terms of exciton scattering, as an electron-hole pair with zero center-of-mass momentum, $(c, k)(v, k)$, has a well-defined chirality with respect to the noninteracting ground state, i.e., $\Delta\mathcal{C} = +2 = 1 - (-1)$ for $k >$ K ($\Delta\mathcal{C} = -2$ for $k <$ K). The chirality of the e-h pair is conserved during Coulomb scattering, i.e., as the pair changes its relative momentum from $2k = k - (-k)$ to $2k'$.

Effect of electronic polarization. In order to appreciate the minor differences between $V(k, k')$ and $W^{\rm DFT}(k, k')$ it is convenient to compare the cuts of the maps of Supplementary Fig. 4 along a line $k' = k_0$, as shown in Supplementary Fig. 5 for $k_0 = 0.289(2\pi)/a$ (panel a) and $0.28(2\pi)/a$ (panel b), respectively. For small momentum transfer, $q = k - k_0 \approx 0$, $V(k, k_0)$ (squares) exhibits a sharper spike than $W^{\rm DFT}(k, k_0)$ (filled circles). This is an effect of the regularization of the singularitity occurring in the DFT approach, as in the first-principles calculation the tube is actually three-dimensional. As $|q|$ increases, V is systematically blushifted with respect to $W^{\rm DFT}$ since it does not take into account the effect of the RPA polarization, $\Pi(q)$, which acquires a finite value.

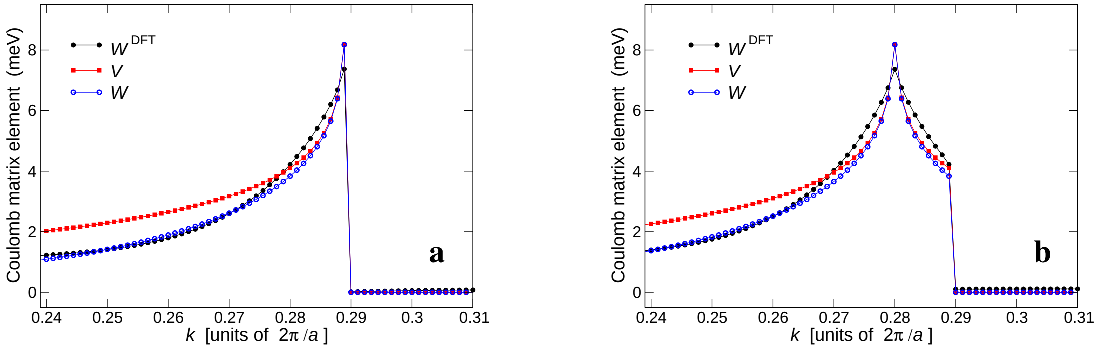

Supplementary Fig. 5 Dominant interband Coulomb matrix elements vs k. Dominant interband Coulomb matrix elements $V(k,k_0)$ (squares), $W(k,k_0)$ (empty circles), and $W^{\rm DFT}(k,k_0)$ (filled circles) vs k, with fixed k_0. **a** $k_0 = 0.289(2\pi)/a$. **b** $k_0 = 0.28(2\pi)/a$. Curves are discontinuous at $\rm K = 0.289(2\pi)/a$, lines are guides to the eye, $N = 900$.

Within the effective-mass approximation, $\Pi(q)$ enters the dressed matrix element W through the dielectric function[13],

$$\varepsilon(q) = 1 + \frac{2e^2}{\kappa_{\rm r}} I_0(R\,|q|)\; K_0(R\,|q|)\; \Pi(q). \tag{19}$$

Here we use the simple ansatz

$$\Pi(q) = A_{\rm ansatz}(Rq)^2, \tag{20}$$

as this choice makes the dressed Coulomb interaction scale like the three-dimensional bare Coulomb potential for large q (i.e., at short distances), $W \sim 1/q^2$. In Supplementary Fig. 5a, b the dressed matrix element W [empty circles, $A_{\rm ansatz} = 50/(\pi\gamma)$, $\gamma/a = 1.783$ eV] quantitatively agrees with its ab initio counterpart, $W^{\rm DFT}$ (filled circles), in the whole range of k in which electrons are massless (cf. Supplementary Fig. 2). Note that for $k > \rm K = 0.289(2\pi)/a$ the effective-mass potentials

are exactly zero whereas $W^{\rm DFT}$ shows some numerical noise.

Effect of the magnetic field. The magnetic field along the tube axis adds an Aharonov-Bohm phase to the transverse momentum, $k_\perp$. This breaks the chiral symmetry $\mathcal{C}$ of single-particle states, alters the form factors of Supplementary Eq. (14) (see Ando[13]), and lifts the selection rule on k. This is apparent from the smearing of the maps of Supplementary Fig. 6 close to the frontiers of the quadrants, $k, k' = $ K, wheres at the same locations in Supplementary Fig. 4 (no field) the plots exhibit sharp discontinuities. The cuts of Supplementary Fig. 6 along the line $k' = k_0$, as shown in Supplementary Figs. 7a and b for $k_0 = 0.289(2\pi)/a$ and $0.28(2\pi)/a$, respectively, confirm the good agreement between $W(k, k_0)$ and $W^{\rm DFT}(k, k_0)$.

Supplementary Note 3

Effective mass: Bethe-Salpeter equation

In this Note we detail the calculation of low-lying excitons of armchair carbon nanotubes, $|\mathrm{u}\rangle$, within the effective mass theory. The analysis of the first-principles exciton wave function for the (3,3) tube shows that the lowest conduction and highest valence bands contribute more than 99.98% to the spectral weight of excitons. Therefore, according to conventional taxonomy, these excitons are of the M_{00} type. Within the effective-mass approximation, $|\mathrm{u}\rangle$ is written as

$$|\mathrm{u}\rangle = \sum_{\sigma\sigma'\tau k} \psi_\tau(k)\, \chi_{\sigma\sigma'}\, \hat{c}^{\tau +}_{k,\sigma}\, \hat{v}^{\tau}_{k,\sigma'} |0\rangle \,, \tag{21}$$

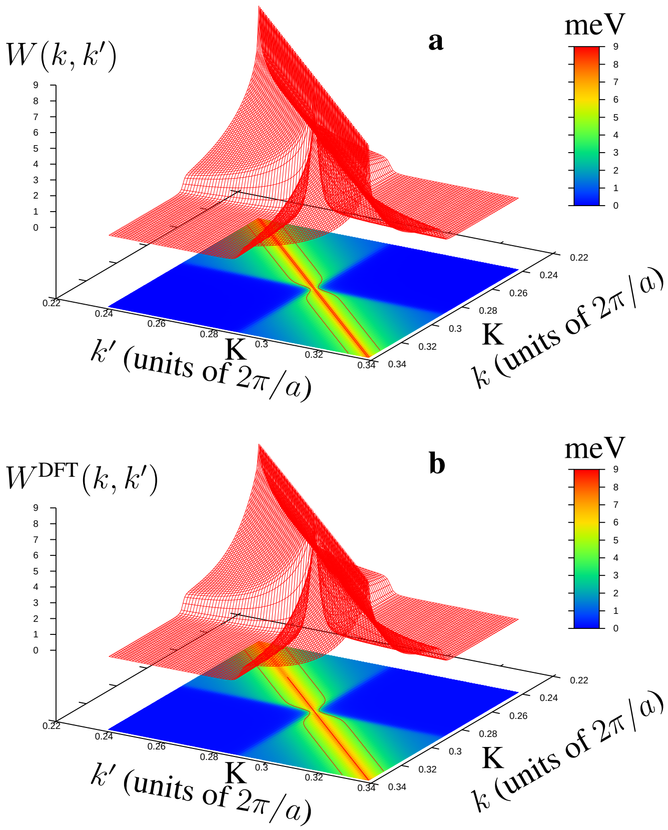

Supplementary Fig. 6 Interband Coulomb matrix element in the (k, k') space in the presence of a magnetic field. Dominant interband Coulomb matrix element in the (k, k') space close to the K point in the presence of a magnetic field, with $\varphi = 7.59 \cdot 10^{-3}$. **a** Effective-mass dressed matrix element $W(k, k')$, with $\kappa_{\rm r} = 10$ and $A_{\rm ansatz} = 50/(\pi\gamma)$. The isolines of the two-dimensional contour map point to the heights of 4 and 8 meV, respectively. **b** Modulus of DFT screened matrix element $W^{\rm DFT}(k, k')$ obtained within the random phase approximation. Here $N = 900$ and K = $0.289(2\pi)/a$.

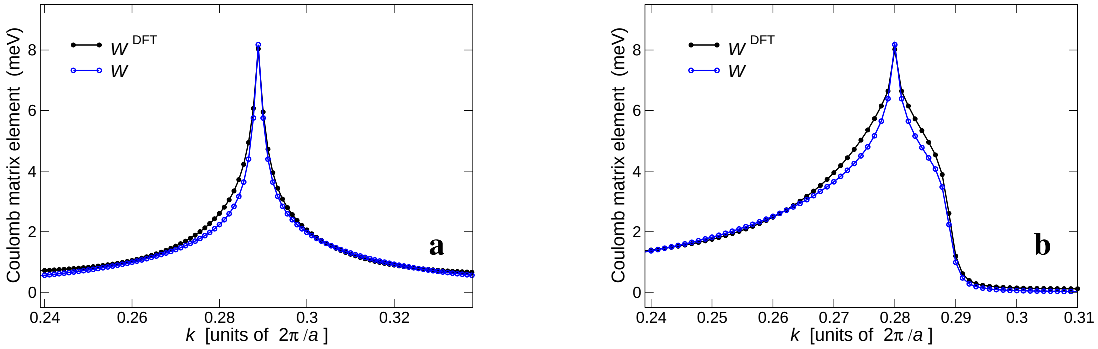

Supplementary Fig. 7 Coulomb matrix element vs k in the presence of the magnetic field. Dominant interband Coulomb matrix elements $W(k, k_0)$ (empty circles) and $W^{\text{DFT}}(k, k_0)$ (filled circles) vs k with fixed k_0 and $\varphi = 7.59 \cdot 10^{-3}$. **a** $k_0 = 0.289(2\pi)/a$. **b** $k_0 = 0.28(2\pi)/a$. Lines are guides to the eye. $N = 900$.

where $|0\rangle$ is the noninteracting ground state with all valence states filled and conduction states empty, and the operator $\hat{c}^{\tau+}_{k,\sigma}$ ($\hat{v}^{\tau+}_{k,\sigma}$) creates an electron in the conduction (valence) band labeled by wave vector k, spin σ, valley τ. The exciton $|\text{u}\rangle$ is a coherent superposition of electron-hole pairs having zero center-of-mass momentum and amplitude $\psi_\tau(k)$. The latter may be regarded as the exciton wave function in k space. The 2×2 spin matrix $\chi_{\sigma\sigma'}$ is the identity for singlet excitons, $\boldsymbol{\chi} = \mathbf{1}_s$, whereas for triplet excitons $\boldsymbol{\chi} = \boldsymbol{\sigma}_s \cdot \mathbf{n}$, where $\mathbf{n}$ is the arbitrary direction of the spin polarization ($|\mathbf{n}| = 1$) and $\boldsymbol{\sigma}_s$ is a vector made of the three Pauli matrices. Throughout this work we ignore the small Zeeman term coupling the magnetic field with electron spin, hence triplet excitons exhibit three-fold degeneracy. Here we use the same notation, $|\text{u}\rangle$, for both singlet and triplet excitons, as its meaning is clear from the context.

The Bethe-Salpeter equation for the triplet exciton is

$$\begin{aligned} & E_{\rm eh}(\tau,k)\,\psi_\tau(k) - \frac{1}{A}\sum_q \tilde{W}^\tau(k+q,k)\;\psi_\tau(k+q) \\ - \quad & \frac{1}{A}\sum_{\tau'\neq\tau}\sum_q \tilde{W}^{\tau\tau'}(k+q,k)\;\psi_{\tau'}(k+q) = \varepsilon_{\rm u}\,\psi_\tau(k). \end{aligned} \tag{22}$$

The diagonal term $E_{\rm eh}(\tau,k)$ is the energy cost to create a free electron-hole pair $(\tau,c,k)(\tau,v,k)$,

$$E_{\rm eh}(\tau,k) = 2\gamma\sqrt{k_\perp^2+k^2} + \Sigma^\tau(k), \tag{23}$$

including the sum of self-energy corrections to electron and hole energies, $\Sigma^\tau(k)$, which may be evaluated e.g. within the GW approximation. This self-energy, which describes the dressing of electrons by means of the interaction with the other electrons present in the tube, is responsible for the small asymmetry of the Dirac cone close to K, as shown by the GW dispersion of Supplementary Fig. 2a. Since this asymmetry appears already at the DFT level of theory and is similar to the one predicted for the Dirac cones of graphene[14], it necessarily originates from mean-field electron-electron interaction and it does not depend on R. We take into account the effect of $\Sigma^\tau(k)$ onto $E_{\rm eh}(\tau,k)$ by explicitly considering different velocities (slopes of the linear dispersions) for respectively left- and right-moving fermions, according to:

$$\begin{aligned} E_{\rm eh}(\mathrm{K},k) &= 2\gamma\left[1+\alpha_{\rm sl}\,\mathrm{sign}(k)\right]\sqrt{k_\perp^2+k^2}, \\ E_{\rm eh}(\mathrm{K}',k) &= 2\gamma\left[1-\alpha_{\rm sl}\,\mathrm{sign}(k)\right]\sqrt{k_\perp^2+k^2}. \end{aligned} \tag{24}$$

We infer the actual values of γ and slope mismatch parameter $\alpha_{\rm sl}$ from the linear fit to the first-principles GW dispersion (in Supplementary Fig. 2b the solid lines are the fits and the dots the GW data), which provides $\gamma = 5.449$ eV·Å and $\alpha_{\rm sl} = 0.05929$.

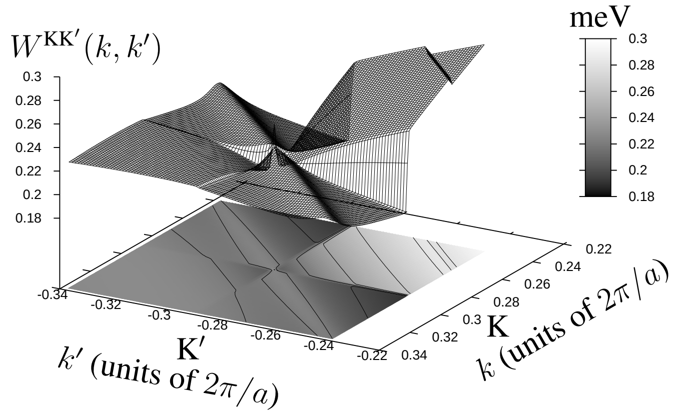

Supplementary Fig. 8 DFT intervalley interband Coulomb matrix element $W^{\mathrm{KK}'}(k,k')$ in (k,k') space. Here $\varphi = 1.52 \cdot 10^{-3}$, $N = 900$, $A = aN$, and K = $= -\mathrm{K}' = 0.289(2\pi)/a$.

The second and third terms on the left hand side of Supplementary Eq. (22) involve interband Coulomb matrix elements. The intravalley term $\tilde{W}^{\tau}$ is the dressed long-ranged interaction discussed in the previous Note. The intervalley term $\tilde{W}^{\mathrm{KK}'}$ makes electron-hole pairs to hop between valleys. As illustrated by the DFT map of $W^{\mathrm{KK}'}(k,k') = \tilde{W}^{\mathrm{KK}'}/A$ in (k,k') space (Supplementary Fig. 8), this term, almost constant in reciprocal space, is at least one order of magnitude smaller than W, as seen by comparing the small range 0.18–0.3 meV of the energy axis of Supplementary Fig. 8 with the range 0–9 meV of Supplementary Figs. 4 and 6. Therefore, $W^{\mathrm{KK}'}$ may be regarded as a weak contact interaction that couples the valleys, consistently with the model by Ando[3,9],

$$\tilde{W}^{\mathrm{KK}'}(k,k') = \frac{\Omega_0 w_2}{4\pi R}, \tag{25}$$

where $\Omega_0 = (\sqrt{3}/2)a^2$ is the area of graphene unit cell and $w_2 > 0$ is the characteristic energy associated with short-range Coulomb interaction. We reasonably reproduce first-principles results taking $w_2 = 2.6$ eV—this would be a plane located at 0.24 meV in Supplementary Fig. 8. This

estimate is not far from Ando's prediction $w_2 = 4$ eV. Note that the previous theory proposed by one of us[3] relies on the scenario $W^{\mathrm{KK}'} > W$, which is ruled out by the present study.

The Bethe-Salpeter equation for the singlet exciton is obtained from Supplementary Eq. (22) by simply adding to the kernel the bare exchange term

$$+ \quad \frac{\Omega_0 w_1}{2\pi R A} \sum_{\tau'} \sum_{q} \psi_{\tau'}(k+q), \tag{26}$$

where $w_1 > 0$ is a characteristic exchange energy[3,9]. From first-principles results we estimate $w_1 = 4.33$ eV, whose magnitude is again comparable to that predicted by Ando[9]. Supplementary Eq. (22), with or without the exchange term, is solved numerically by means of standard linear algebra routines.

Minimal Bethe-Salpeter equation. The minimal Bethe-Salpeter equation illustrated in the main text includes only one valley (with $\alpha_{\mathrm{sl}} = 0$) and long-range Coulomb interaction. Within the effective-mass approximation, the Dirac cone indefinitely extends in momentum space, hence one has to introduce a cutoff onto allowed momenta, $|k| \leq k_{\mathrm{c}}$. Supplementary Fig. 9a shows the convergence of the lowest-exciton energy, ε_{u}, as a function of k_{c}. Reassuringly, ε_{u} smoothly converges well within the range in which GW bands are linear. This is especially true for the screened interaction W (black circles), whereas the convergence is slower for the unscreened interaction V (red circles), as it is obvious since $W(q)$ dies faster with increasing q. This behavior implies that the energy scale associated with the exciton is intrinsic to the tube and unrelated to the cutoff, as we further discuss below.

In the reported calculations we took $k_{\mathrm{c}} = 0.05(2\pi)/a$ as a good compromise between accuracy and computational burden (we expect that the maximum absolute error on ε_{u} is less than 0.1 meV). This corresponds to an energy cutoff of 1.4 eV for e-h pair excitations. Whereas for these calculations, as well as for the data of Supplementary Fig. 9a, the mesh Δk in momentum space is fixed [$\Delta k = 1.43 \cdot 10^{-5}(2\pi)/a$], Supplementary Fig. 9b shows the convergence of ε_{u} as a function of the mesh, Δk. Interestingly, ε_{u} smoothly decreases with Δk only for a very fine mesh, whereas for larger values of Δk the energy exhibits a non-monotonic behaviour. This is a consequence of the logarithmic spike of the Colulomb potential at vanishing momentum, which requires a very fine mesh to be dealt with accurately.

We refine the minimal effective-mass Bethe-Salpeter equation by including: (i) The short-range part of interaction, which couples the two valleys as well as lifts the degeneracy of spin singlet and triplet excitons. (ii) The tiny difference between the e-h pair excitation energies of left and right movers. This eventually leads to a quantitative agreement with exciton energies and wave functions obtained from first principles, as shown by Fig. 3b, c and Fig. 6a, b of main text.

Scaling properties of the Bethe-Salpeter equation. If a well-defined (i.e., bound and normalizable) solution of the Bethe-Salpeter equation (22) exists, then it must own a characteristic length and energy scale—respectively the exciton Bohr radius and binding energy[15]. To check this, we introduce the scaling length ℓ to define the following dimensionless quantities: the wave vector $\kappa = k\ell$, the energy $\mathcal{E}_{\mathrm{u}} = \varepsilon_{\mathrm{u}}\ell/\gamma$, and the exciton wave function $\xi_\tau(\kappa) = \psi_\tau(k)/\sqrt{\ell}$. We also define the dimensionless intravalley interaction as $\Omega^\tau(kR, k'R) = (\kappa_{\mathrm{r}}/e^2)\tilde{W}^\tau(k, k')$, to highlight

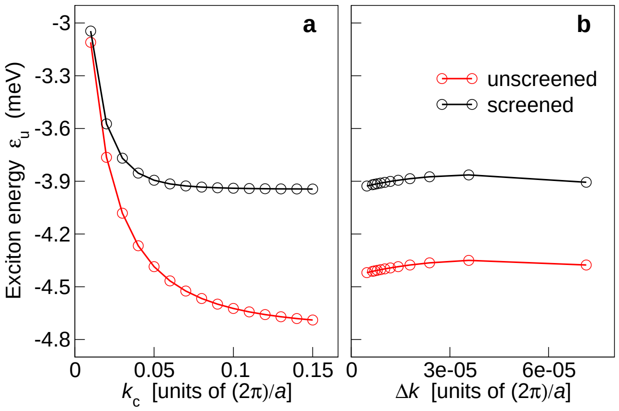

Supplementary Fig. 9 Convergence of exciton energy within a single valley in the effective-mass approximation. **a** Excitation energy of the lowest exciton, ε_{u}, vs cutoff in momentum space, k_{c}. The black (red) curve is the energy obtained using the screened (unscreened) long-range interaction, W (V), in the Bethe-Salpeter equation for the triplet exciton. Here $\Delta k = 1.43 \cdot 10^{-5}(2\pi)/a$ and $\varphi = 1.52 \cdot 10^{-5}$. **b** Excitation energy of the lowest exciton, ε_{u}, vs mesh in momentum space, Δk. Here $k_{\mathrm{c}} = 0.05(2\pi)/a$ and $\varphi = 1.52 \cdot 10^{-5}$.

that the wave vector k appearing as an argument of the interaction is always multiplied by R. This is important for the exciton scaling behaviour.

Neglecting the small corrections to the exciton binding energy due to intervalley scattering ($w_2 = 0$) and cone asymmetry ($\alpha_{\text{sl}} = 0$), the dimensionless Bethe-Salpeter equation for armchair tubes in the absence of a magnetic flux becomes

$$2\,|\kappa|\,\xi_\tau(\kappa) \;-\; \frac{\alpha_{\text{graph}}}{2\pi}\int d\kappa'\;\Omega^\tau[\kappa'(R/\ell),\kappa(R/\ell)]\;\xi_\tau(\kappa') = \mathcal{E}_{\text{u}}\;\xi_\tau(\kappa), \tag{27}$$

where $\alpha_{\text{graph}} = e^2/(\kappa_{\text{r}}\gamma)$ is graphene fine-structure constant, the scaled exciton wave function must satisfy the scale invariant normalization requirement, $\sum_\tau \int d\kappa\,|\xi_\tau(\kappa)|^2 = 1$, and the dielectric function entering Ω takes the dimensionless form

$$\varepsilon(\kappa) = 1 + \frac{2A_{\text{ansatz}}}{\pi}\alpha_{\text{graph}}\kappa^2(R/\ell)^2 I_0(|\kappa|\,R/\ell)\;K_0(|\kappa|\,\ell/R)\,. \tag{28}$$

The only scaling length ℓ leaving Supplementary Eqs. (27) and (28) invariant is the tube radius, R, wich fixes the binding energy unit, γ/R. Supplementary Eq. (27) shows that α_{graph} is the single parameter combination affecting the scale invariant solution, whereas solutions for different radius R are related via scaling,

$$\varepsilon_{\text{u}} = \frac{E_0}{R}, \tag{29}$$

with E_0 being calculated once for all for the (3,3) tube radius, $R = 2$ Å. The same conclusion holds for finite cone asymmetry α_{sl} and dimensionless magnetic flux φ. Note that, for a fixed value of φ, the possible values of the magnetic field B scale like $1/R^2$.

The above demonstration relies on the assumption that the parameters κ_{r} and A_{ansatz}, which

control the screening behavior of the carbon nanotube, do not depend significantly on R. On the other hand, one might expect to recover, for large R, the screening properties of graphene. This in turn would imply that κ_{r} would tend to smaller values and hence ε_{u} would decay slower than $1/R$. The first-principles investigation of this issue is left to future work.

Supplementary Note 4

Self-consistent mean-field theory of the excitonic insulator

The ground-state wave function of the excitonic insulator, $|\Psi_{\mathrm{EI}}\rangle$, exhibits a BCS-like form,

$$|\Psi_{\mathrm{EI}}\rangle = \prod_{\sigma\sigma'\tau k} \left[u_{\tau k} + \chi_{\sigma\sigma'} v_{\tau k} e^{i\eta}\, \hat{c}^{\tau +}_{k,\sigma} \hat{v}^{\tau}_{k,\sigma'} \right] |0\rangle \,, \tag{30}$$

where η is the arbitrary phase of the condensate, the e-h pairs $\hat{c}^{\tau+}_{k,\sigma}\hat{v}^{\tau}_{k,\sigma'}|0\rangle$ replace the Cooper pairs (e.g. $\hat{c}^{\mathrm{K}+}_{k,\sigma}\hat{c}^{\mathrm{K}'+}_{-k,-\sigma}|0\rangle$), and the 2×2 matrix $\chi_{\sigma\sigma'}$ discriminates between singlet and triplet spin symmetries. The positive variational quantities $u_{\tau k}$ and $v_{\tau k}$ are the population amplitudes of valence and conduction levels, respectively, which are determined at once with the excitonic order parameter, $\Delta(\tau k)$. Explicitly, one has

$$\begin{aligned} u^2_{\tau k} &= \frac{1}{2}\left(1 + \frac{E_{\mathrm{eh}}(\tau,k)/2}{\left[E^2_{\mathrm{eh}}(\tau,k)/4 + |\Delta(\tau k)|^2\right]^{1/2}} \right), \\ v^2_{\tau k} &= 1 - u^2_{\tau k}, \end{aligned} \tag{31}$$

plus the self-consistent equation for Δ [equivalent to Eq. (2) of main text],

$$|\Delta(\tau k)| = \frac{1}{A}\sum_{\tau' k'} \tilde{W}^{\tau\tau'}(k,k') \frac{|\Delta(\tau' k')|}{2\left[E^2_{\mathrm{eh}}(\tau',k')/4 + |\Delta(\tau' k')|^2\right]^{1/2}}. \tag{32}$$

The symbol $\tilde{W}^{\tau\tau'}(k,k')$ in Supplementary Eq. (32) is a shorthand for both intra and intervalley Coulomb interaction matrix elements. For the spin-triplet EI [$\chi_{\sigma\sigma'} = (\boldsymbol{\sigma}_s \cdot \mathbf{n})_{\sigma\sigma'}$], which is the absolute ground state, one has, for $\tau = \tau'$, the long-range intravalley term, $\tilde{W}^{\tau\tau}(k,k') = \tilde{W}^{\tau}(k,k')$, and, for $\tau \neq \tau'$, the short-range intervalley term, $\tilde{W}^{\tau\tau'}(k,k')$. For the spin singlet ($\chi_{\sigma\sigma'} = \delta_{\sigma\sigma'}$), the unscreened direct term must be subtracted from the dressed interaction, $\tilde{W}^{\tau\tau'}(k,k') \to \tilde{W}^{\tau\tau'}(k,k') - \Omega_0 w_1/2\pi R$. Supplementary Eq. (32) allows for a scaling analysis similar to that for the exciton binding energy.

If interaction matrix elements $\tilde{W}$ were constant, then Supplementary Eq. (32) would turn into the familiar gap equation of BCS theory, with Δ constant as well. Since the long-range part of interaction is singular, the dependence of $\Delta(\tau k)$ on τ and k cannot be neglected and hence the solution is not obvious. It is convenient to rewrite Supplementary Eq. (32) as a pseudo Bethe-Salpeter equation,

$$2\left[E_{\text{eh}}^2(\tau,k)/4 + |\Delta(\tau k)|^2\right]^{1/2} \varphi(\tau k) - \frac{1}{A}\sum_{\tau' k'} \tilde{W}^{\tau\tau'}(k,k')\, \varphi(\tau' k') = 0, \tag{33}$$

with the pseudo exciton wave function defined as

$$\varphi(\tau k) = \frac{|\Delta(\tau k)|}{2\left[E_{\text{eh}}^2(\tau,k)/4 + |\Delta(\tau k)|^2\right]^{1/2}}. \tag{34}$$

This shows that, at the onset of the EI phase, when $\Delta(\tau k)$ is infinitesimal—at the critical magnetic field—the exciton wave function for $\varepsilon_{\text{u}} = 0$ is the same as φ apart from a constant, $\varphi(\tau k) \sim \psi_\tau(k)$. This observation suggests to use $\psi_\tau(k)$ at all values of the field as a good ansatz to start the self-consistent cycle of Supplementary Eq. (33), which is numerically implemented as a matrix product having the form $\boldsymbol{\varphi}_{\text{new}} = \boldsymbol{W} \cdot \boldsymbol{\varphi}_{\text{old}}$. Taking at the first iteration $|\Delta_{\text{old}}(\tau k)| =$

$2^{-1}\left[E_{\rm eh}(\tau,k)-\varepsilon_{\rm u}\right]\left|\psi_\tau(k)/\psi_\tau(0)\right|$ and building $\varphi_{\rm old}(\tau k)$ according to Supplementary Eq. (34), we obtain numerical convergence within a few cycles, $\boldsymbol{\varphi}_{\rm new}=\boldsymbol{\varphi}_{\rm old}$, with the number of iterations increasing with decreasing Δ. At finite temperatures, the self-consistent equation for Δ takes the form

$$\begin{aligned} |\Delta(\tau k)| &= \frac{1}{A}\sum_{\tau' k'} \tilde{W}^{\tau\tau'}(k,k') \frac{|\Delta(\tau' k')|}{2\left[E^2_{\rm eh}(\tau',k')/4+|\Delta(\tau' k')|^2\right]^{1/2}} \\ &\times \ \tanh\left\{\frac{1}{2k_{\rm B}T}\left[E^2_{\rm eh}(\tau',k')/4+|\Delta(\tau' k')|^2\right]^{1/2}\right\}, \end{aligned} \tag{35}$$

where T is the temperature and $k_{\rm B}$ is Boltzmann constant.

The quasiparticles of the EI are the free electrons and holes. For example, in the simplest case of the spin-singlet EI ($\chi_{\sigma\sigma'}=\delta_{\sigma\sigma'}$), the electron quasiparticle wave function $\left|\Psi^{\tau k\uparrow}_{\rm EI}\right\rangle$ differs from the ground state $|\Psi_{\rm EI}\rangle$ as the conduction electron state labeled by $(\tau,k,\uparrow)$ is occupied with probability one as well as the corresponding valence state:

$$\left|\Psi^{\tau k\uparrow}_{\rm EI}\right\rangle = \hat{c}^{\tau +}_{k,\uparrow}\left[u_{\tau k}+v_{\tau k}e^{i\eta}\,\hat{c}^{\tau +}_{k,\downarrow}\hat{v}^{\tau}_{k,\downarrow}\right]\prod_{\sigma\tau' k'}{}^{'}\left[u_{\tau' k'}+v_{\tau' k'}e^{i\eta}\,\hat{c}^{\tau' +}_{k',\sigma}\hat{v}^{\tau'}_{k',\sigma}\right]|0\rangle\,. \tag{36}$$

Here the symbol $\prod'$ means that the dummy indices $\tau' k'$ take all values but τk. The quasiparticle energy dispersion is

$$E(\tau k)=\sqrt{E^2_{\rm eh}(\tau,k)/4+|\Delta(\tau k)|^2}\,, \tag{37}$$

with the reference chemical potential being zero, as for the noninteracting undoped ground state. $E(\tau k)$ is increased quadratically by the amount $|\Delta(\tau k)|$ with respect to the noninteracting energy, $\varepsilon(\tau k)=E_{\rm eh}(\tau,k)/2$. This extra energy cost is a collective effect reminescent of the exciton binding energy, since now the exciton condensate must be ionized to unbind one e-h pair and hence have a free electron and hole.

Supplementary Note 5

Inversion symmetry breaking in the excitonic insulator phase

Carbon nanotubes inherit from graphene fundamental symmetries such as time reversal and spatial inversion. Time reversal $\hat{\mathbb{T}}$ swaps K and K$'$ valleys whereas the inversion $\hat{\mathbb{I}}$ is a π rotation around an axis perpendicular to the tube surface and located in the origin of one of the frames shown in Supplementary Fig. 1. This swaps the valleys as well as the A and B sublattices. Whereas the noninteracting ground state $|0\rangle$ is invariant under both inversion and time reversal, $\hat{\mathbb{T}}\,|0\rangle = |0\rangle$ and $\hat{\mathbb{I}}\,|0\rangle = |0\rangle$, the EI ground state breaks the inversion symmetry[16]. Here we consider a spin-singlet exciton condensate ($\chi_{\sigma\sigma'} = \delta_{\sigma\sigma'}$) with $\hat{\mathbb{T}}\ \ |\Psi_{\text{EI}}\rangle = |\Psi_{\text{EI}}\rangle$, hence the excitonic order parameter is real, $\eta = 0, \pi$ (otherwise the EI ground state would exhibit transverse orbital currents).

To see that the inversion symmetry of the EI ground state is broken we use the following transformations (whose details are given in Supplementary Note 7):

$$
\begin{aligned}
\hat{\mathbb{I}}\; \hat{v}^{\tau}_{k,\sigma} &= -i\, \text{sign}(k)\, \hat{v}^{-\tau}_{-k,\sigma}, \\
\hat{\mathbb{I}}\; \hat{c}^{\tau}_{k,\sigma} &= i\, \text{sign}(k)\, \hat{c}^{-\tau}_{-k,\sigma},
\end{aligned} \tag{38}
$$

where the shorthand $-\tau$ labels the valley other than τ. The transformed ground state is

$$
\hat{\mathbb{I}}\ \ |\Psi_{\text{EI}}\rangle = \prod_{\sigma\tau k} \left[u_{\tau k} - v_{\tau k} e^{i\eta}\, \hat{c}^{\tau +}_{k,\sigma} \hat{v}^{\tau}_{k,\sigma} \right] |0\rangle\,, \tag{39}
$$

where we have used the fact that $u_{\tau k} = u^{*}_{\tau k} = u_{-\tau -k}$ and $v_{\tau k} = v^{*}_{\tau k} = v_{-\tau -k}$, as a consequence of time reversal symmetry. The original and transformed ground states are orthogonal in the thermo-

dynamic limit,

$$\langle \Psi_{\mathrm{EI}} | \,\hat{\mathbb{I}}\, | \Psi_{\mathrm{EI}} \rangle = 2 \prod_{\tau k} \left(u_{\tau k}^2 - v_{\tau k}^2 \right) \to 0, \tag{40}$$

since $u^2 - v^2 < 1$. On the contrary, $\langle 0 | \,\hat{\mathbb{I}}\, | 0 \rangle = 1$. Therefore, the symmetry of the EI ground state is lower than that of the noninteracting ground state so the EI phase has broken inversion symmetry, i.e., charge is displaced from A to B sublattice or vice versa.

Supplementary Note 6

Charge displacement between A and B sublattices

In this section we compute the charge displacement between A and B carbon sublattices in the EI ground state. To this aim we must average over the ground state the space-resolved charge density

$$\varrho(\mathbf{r}) = e \sum_i \delta(\mathbf{r} - \mathbf{r}_i), \tag{41}$$

where the sum runs over all electrons in the Dirac valleys. The explicit form of the charge density, in second quantization, is

$$\begin{aligned} \hat{\varrho}(\mathbf{r}) &= e \sum_{\tau k \tau' k' \sigma} \Big[\varphi^*_{c\tau k}(\mathbf{r}) \varphi_{c\tau' k'}(\mathbf{r}) \hat{c}^{\tau +}_{k,\sigma} \hat{c}^{\tau'}_{k',\sigma} + \varphi^*_{v\tau k}(\mathbf{r}) \varphi_{v\tau' k'}(\mathbf{r}) \hat{v}^{\tau +}_{k,\sigma} \hat{v}^{\tau'}_{k',\sigma} \\ &+ \varphi^*_{c\tau k}(\mathbf{r}) \varphi_{v\tau' k'}(\mathbf{r}) \hat{c}^{\tau +}_{k,\sigma} \hat{v}^{\tau'}_{k',\sigma} + \varphi^*_{v\tau k}(\mathbf{r}) \varphi_{c\tau' k'}(\mathbf{r}) \hat{v}^{\tau +}_{k,\sigma} \hat{c}^{\tau'}_{k',\sigma} \Big]. \end{aligned} \tag{42}$$

We recall that the states of conduction ($\alpha = c$) and valence ($\alpha = v$) bands appearing in Supplementary Eq. (42), $\varphi_{\alpha\tau k}(\mathbf{r})$, are products of the envelope functions F times the Bloch states ψ_τ at Brillouin zone corners $\tau = \mathrm{K}, \mathrm{K}'$,

$$\varphi_{\alpha\tau k}(\mathbf{r}) = F^{\tau A}_{\alpha k}(\mathbf{r})\, \psi_{\tau A}(\mathbf{r}) + F^{\tau B}_{\alpha k}(\mathbf{r})\, \psi_{\tau B}(\mathbf{r}), \tag{43}$$

where $\psi_{\tau A}(\mathbf{r})$ [$\psi_{\tau B}(\mathbf{r})$] is the component on the A (B) sublattice. Neglecting products of functions localized on different sublattices, like $\psi^*_{\tau A}\psi_{\tau B}$, as well as products of operators non diagonal in τ and k indices, which are immaterial when averaging over the ground state, one obtains:

$$\begin{aligned}\hat{\varrho}(\mathbf{r}) &= \frac{e}{2AL}\sum_{\tau}\left[|\psi_{\tau A}(\mathbf{r})|^2+|\psi_{\tau B}(\mathbf{r})|^2\right]\sum_{k\sigma}\left(\hat{v}^{\tau+}_{k,\sigma}\hat{v}^{\tau}_{k,\sigma}+\hat{c}^{\tau+}_{k,\sigma}\hat{c}^{\tau}_{k,\sigma}\right)\\ &+ \frac{e}{2AL}\sum_{\tau}\left[|\psi_{\tau A}(\mathbf{r})|^2-|\psi_{\tau B}(\mathbf{r})|^2\right]\sum_{k\sigma}\left(\hat{c}^{\tau+}_{k,\sigma}\hat{v}^{\tau}_{k,\sigma}+\hat{v}^{\tau+}_{k,\sigma}\hat{c}^{\tau}_{k,\sigma}\right).\end{aligned} \tag{44}$$

The first and second line on the right hand side of Supplementary Eq. (44) are respectively the intra and interband contribution to the charge density. Only the intraband contribution survives when averaging $\hat{\varrho}$ over $|0\rangle$, providing the noninteracting system with the uniform background charge density $\varrho_0(\mathbf{r})$,

$$\varrho_0(\mathbf{r}) = \langle 0|\,\hat{\varrho}(\mathbf{r})\,|0\rangle = \frac{e}{aL}\sum_{\tau}\left[|\psi_{\tau A}(\mathbf{r})|^2+|\psi_{\tau B}(\mathbf{r})|^2\right], \tag{45}$$

with $\sum_k 1 = A/a$. Since $|\psi_{\mathrm{K}A}(\mathbf{r})| = |\psi_{\mathrm{K}'A}(\mathbf{r})| = |\psi_A(\mathbf{r})|$, and similarly for B, this expression may be further simplified as

$$\varrho_0(\mathbf{r}) = \frac{2e}{aL}\left[|\psi_A(\mathbf{r})|^2+|\psi_B(\mathbf{r})|^2\right]. \tag{46}$$

It is clear from this equation that ϱ_0 is obtained by localizing the two π-band electrons uniformly on each sublattice site. When averaging $\hat{\varrho}$ over $|\Psi_{\mathrm{EI}}\rangle$, the charge density $\varrho(\mathbf{r})$ exhibits an additional interband contribution,

$$\varrho(\mathbf{r}) = \langle\Psi_{\mathrm{EI}}|\,\hat{\varrho}(\mathbf{r})\,|\Psi_{\mathrm{EI}}\rangle = \varrho_0(\mathbf{r}) + \frac{2e\cos\eta}{AL}\left[|\psi_A(\mathbf{r})|^2-|\psi_B(\mathbf{r})|^2\right]\sum_{\tau k}u_{\tau k}v_{\tau k}, \tag{47}$$

which is proportional to $\sum_{\tau k}u_{\tau k}v_{\tau k}$ and hence related to the EI order parameter. This term, whose origin is similar to that of the transition density shown in Fig. 3d of main text, as it takes into

account the polarization charge fluctuation between $|0\rangle$ and a state with one or more e-h pairs excited, is driven from the long-range excitonic correlations. Importantly, the charge displacement is uniform among all sites of a given sublattice and changes sign with sublattice, the sign depending on the phase of the exciton condensate, η. The charge displacement per electron, $\Delta e/e$, on—say—each A site is

$$\frac{\Delta e}{e} = \frac{a \cos\eta}{A} \sum_{\tau k} u_{\tau k} v_{\tau k}, \tag{48}$$

which is the same as Eq. (3) of main text. In order to evaluate numerically $\Delta e/e$, for the sake of simplicity we neglect the exchange terms splitting the triplet and singlet order parameters (i.e., we assume $w_1 = 0$). The quantum Monte Carlo order parameter ϱ_{AB} defined in the main text is, in absolute value, twice $|\Delta e/e|$ as there are two relevant electrons per site.

Supplementary Note 7

Reference frame and symmetry operations

The reference frame of the armchair carbon nanotube shown in Supplementary Fig. 1a is obtained by rigidly translating the frame used by Ando in a series of papers[2,9,13], recalled in Supplementary Fig. 1b. In Ando's frame the origin is placed on an atom of the B sublattice and the y axis is parallel to the tube axis, after a rotation by the chiral angle α with respect to the y' axis of graphene. On the basis of primitive translation vectors of graphene $\mathbf{a}$ and $\mathbf{b}$ displayed in Supplementary Fig. 1b, the chiral vector takes the form $\mathbf{L} = -m\mathbf{a} - (n+m)\mathbf{b}$ when expressed in terms of the conventional[17] chiral indices (n, m). For an equivalent choice of $\mathbf{L}$, one has $\alpha = \pi/6$ for (n, n) armchair tubes

and $\alpha = 0$ for $(n, 0)$ zigzag tubes.

In the frame of Supplementary Fig. 1a used throughout this Supplementary Information, the vectors locating the sites of A and B sublattices are respectively

$$\mathbf{R}^A_{n_a,n_b} = \mathbf{R}^A_0 + n_a\mathbf{a} + n_b\mathbf{b} \tag{49}$$

and

$$\mathbf{R}^B_{n_a,n_b} = \mathbf{R}^B_0 + n_a\mathbf{a} + n_b\mathbf{b}, \tag{50}$$

where (n_a, n_b) is a couple of integers and $\mathbf{R}^A_0$ ($\mathbf{R}^B_0$) is the basis vector pointing to the origin of the A (B) sublattice. Besides, one has $\mathbf{a} \equiv a(\sqrt{3}/2, -1/2)$, $\mathbf{b} \equiv a(0, 1)$, $\mathbf{R}^A_0 \equiv a(2/\sqrt{3}, 1/2)$, $\mathbf{R}^B_0 \equiv a(\sqrt{3}/2, 0)$, where $a = 2.46$ Å is the lattice constant of graphene. Among the equivalent corners of graphene first Brillouin zone, we have chosen as Dirac points $\mathbf{K} \equiv \frac{2\pi}{a}(1/\sqrt{3}, 1/3)$ and $\mathbf{K}' = -\mathbf{K}$. The corresponding Bloch states are:

$$\begin{aligned}
\psi_{\mathrm{K}A}(\mathbf{r}) &= \frac{1}{\sqrt{N}}\sum_{n_a,n_b} e^{i\mathbf{K}\cdot\mathbf{R}^A_{n_a,n_b}}\,\phi_\pi(\mathbf{r}-\mathbf{R}^A_{n_a,n_b}),\\
\psi_{\mathrm{K}B}(\mathbf{r}) &= -e^{i\pi/6}\,\omega\frac{1}{\sqrt{N}}\sum_{n_a,n_b} e^{i\mathbf{K}\cdot\mathbf{R}^B_{n_a,n_b}}\,\phi_\pi(\mathbf{r}-\mathbf{R}^B_{n_a,n_b}),\\
\psi_{\mathrm{K}'A}(\mathbf{r}) &= e^{i\pi/6}\,\omega\frac{1}{\sqrt{N}}\sum_{n_a,n_b} e^{i\mathbf{K}'\cdot\mathbf{R}^A_{n_a,n_b}}\,\phi_\pi(\mathbf{r}-\mathbf{R}^A_{n_a,n_b}),\\
\psi_{\mathrm{K}'B}(\mathbf{r}) &= \frac{1}{\sqrt{N}}\sum_{n_a,n_b} e^{i\mathbf{K}'\cdot\mathbf{R}^B_{n_a,n_b}}\,\phi_\pi(\mathbf{r}-\mathbf{R}^B_{n_a,n_b}),
\end{aligned} \tag{51}$$

where N is the number of sublattice sites, $\phi_\pi(\mathbf{r})$ is the $2p_z$ carbon orbital perpendicular to the graphene plane, normalized as in Secchi & Rontani[10], and $\omega = \exp{(i2\pi/3)}$.

The relative phase between the two sublattice components of Bloch states within each valley, shown in Supplementary Eq. (51), is determined by symmetry considerations[18]. Specifically, the

sublattice pseudospinor transforms as a valley-specific irreducible representation of the symmetry point group of the triangle, C_{3v}:

$$\boldsymbol{F}_{\mathrm{K}\alpha k} \sim \begin{pmatrix} x - iy \\ x + iy \end{pmatrix}, \qquad \boldsymbol{F}_{\mathrm{K}'\alpha k} \sim \begin{pmatrix} x + iy \\ -x + iy \end{pmatrix}. \tag{52}$$

In addition, the relative phase between Bloch states of different valleys is fixed by exploiting the additional C_2 symmetry. The latter consists of a rotation of a π angle around the axis perpendicular to the graphene plane and intercepting the frame origin. This rotation, which in the xy space is equivalent to the inversion $\hat{\mathbb{I}}$, swaps K and K′ valleys as well as A and B sublattices. With the choice of phases explicited in Supplementary Eq. (51) the inversion operator $\hat{\mathbb{I}}$ takes the form

$$\hat{\mathbb{I}} = -\boldsymbol{\sigma}_y \otimes \boldsymbol{\tau}_y \, \hat{R}, \tag{53}$$

where $\hat{R}$ is the inversion operator in the xy space. In contrast, the time-reversal operator $\hat{\mathbb{T}}$ swaps valleys but not sublattices,

$$\hat{\mathbb{T}} = \boldsymbol{\sigma}_z \otimes \boldsymbol{\tau}_x \, \hat{K}, \tag{54}$$

where $\hat{K}$ is the complex-conjugation operator. The orthogonal time-reversal of Supplementary Eq. (54) should not be confused with the symplectic transformation[19], which does not exchange valleys.

The magnetic field along the tube axis breaks both $\hat{\mathbb{I}}$ and $\hat{\mathbb{T}}$ symmetries. However, the reflection symmetry $y \to -y$ along the tube axis still swaps the valleys (but not sublattices), as it may be easily seen from a judicious choice of K and K′ Dirac points. This protects the degeneracy of states belonging to different valleys in the presence of a magnetic field.

Supplementary Discussion

Effects of Dirac cone asymmetry and magnetic field on the exciton wave function

The origin of the asymmetry of the exciton wave function in k space, illustrated by Fig. 3b of main text, may be understood within the effective mass model applied to a single Dirac valley—say K. In the presence of a vanishing gap, electrons (and excitons) acquire a chiral quantum number, $\mathcal{C}$, which was defined above. With reference to the noninteracting ground state, $|0\rangle$, the e-h pairs $\hat{c}^{K\dagger}_{k,\sigma}\hat{v}^{K}_{k,\sigma'}\,|0\rangle$ have chiral quantum number $\Delta\mathcal{C} = +2$ for positive k and $\Delta\mathcal{C} = -2$ for negative k. Since long-range Coulomb interaction conserves chirality, we expect the wave function of a chiral exciton to live only on one semi-axis in k space, either $\psi_K(k) = 0$ for $k < 0$ and $\Delta\mathcal{C} = +2$, or $\psi_K(k) = 0$ for $k > 0$ and $\Delta\mathcal{C} = -2$.

Supplementary Fig. 10 plots $\psi_K(k)$ by comparing the case of a perfectly symmetric Dirac cone (panel a, $\alpha_{\text{sl}} = 0$) with the case of a distorted cone, mimicking the first-principles GW band dispersion (panel b, $\alpha_{\text{sl}} = 0.05929$). This analysis is of course possible only within the effective mass model, as no free parameter such as α_{sl} may be changed in the first-principles calculation. In the symmetric case (Supplementary Fig. 10a) $\psi_K(k)$ is even in k since nothing prevents the numerical diagonalization routine from mixing the two degenerate amplitude distributions with $\Delta\mathcal{C} = \pm 2$. Hovever, as the Dirac cone symmetry under axis inversion, $k \rightarrow -k$, is lifted by energetically favoring e-h pairs with $\Delta\mathcal{C} = -2$ (Supplementary Fig. 10b), the wave function weight collapses on the negative side of the axis. Therefore, the asymmetry of the exciton wave function is explained by the combined effects of chiral symmetry and cone distortion.

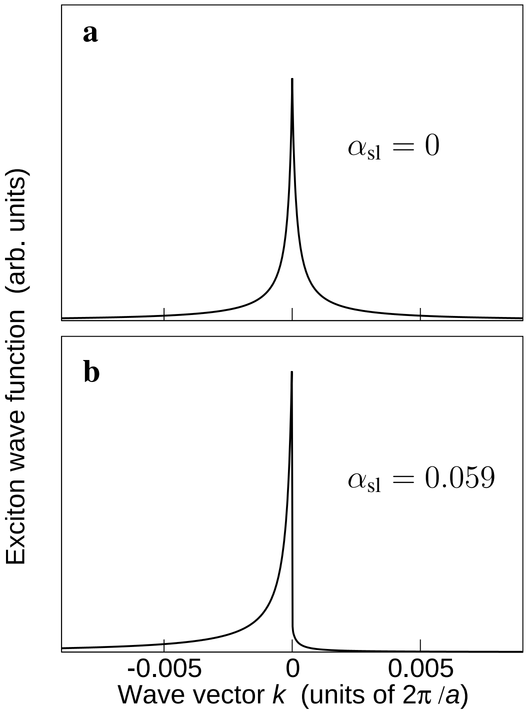

Supplementary Fig. 10 Effect of the asymmetry of the Dirac cone on the exciton wave function within the effective mass approximation. Wave function of the lowest-energy exciton within a single valley, $\psi_{\rm K}(k)$, vs wave vector, k. **a** The slope asymmetry parameter has a vanishing value, $\alpha_{\rm sl} = 0$, hence the Dirac cone is symmetric under axis inversion, $k \to -k$. **b** $\alpha_{\rm sl} = 0.05929$. Here $w_2 = w_1 = 0$ and $\varphi = 1.52 \cdot 10^{-5}$.

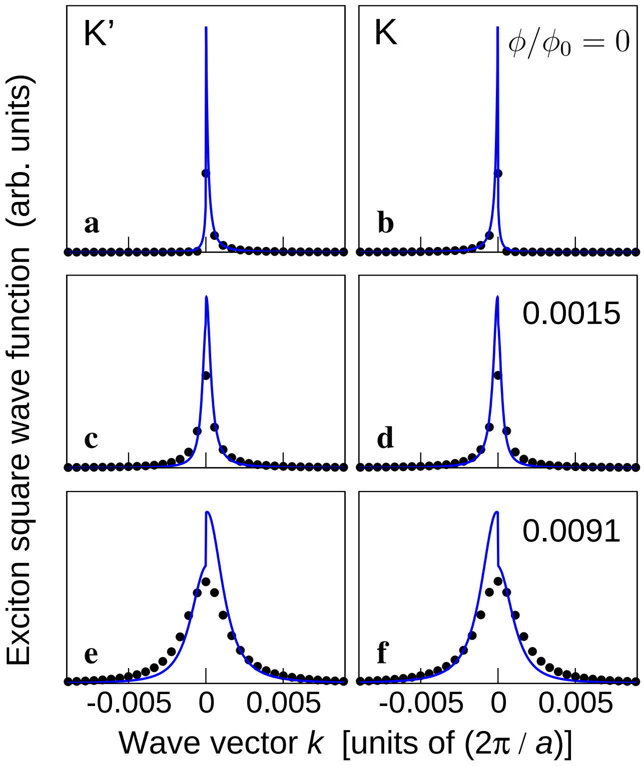

Supplementary Fig. 11 Effect of the magnetic field on the exciton wave function in k space. Square modulus of the wave function of the lowest triplet exciton, $|\psi_\tau(k)|^2$, vs wave vector, k, for increasing values of the magnetic flux, ϕ/ϕ_0. **a, b** $\phi/\phi_0 = 0$. **c, d** $\phi/\phi_0 = 0.0015$. **e, f** $\phi/\phi_0 = 0.0091$. Panels a, c, e (b, d, f) refer to valley K$'$ (K). The first-principles data for the (3,3) tube (black dots) are compared with the effective-mass predictions (blue curves). As the field increases the weight distribution becomes broader and more symmetrical around the Dirac point.

As the chiral symmetry is destroyed by piercing the tube with a magnetic flux, the exciton wave function ψ becomes symmetrically distibuted around the origin of k axis. This is shown in Supplementary Fig. 11 from both first-principles (dots) and effective-mass (solid curves) calculations of $|\psi_\tau(k)|^2$ for increasing values of the dimensionless magnetic flux ϕ/ϕ_0 (for panels a,b $\phi/\phi_0 = 0$, for c, d $\phi/\phi_0 = 0.0015$, for e, f $\phi/\phi_0 = 0.0091$). As the gap increases with the field, the excitons becomes massive and more similar to the conventional Wannier excitons reported in the literature[13,20,21]: the weight distribution in k space is broader and its peak more rounded, with a Gaussian-like shape identical in both valleys (respectively valley K$'$ in panels a, c, e and valley K in panels b, d, f). The agreement between first-principles (dots) and effective-mass (solid lines) predictions is very good, further validating the model. However, at high field (panels e and f, $\phi/\phi_0 = 0.0091$), the effective-mass curve becomes discontinuous at the Dirac cone whereas the first-principles curve is smooth. This is an artefact of the effective-mass model as the high-field functional form of the distorted Dirac cone shown by Supplementary Eq. (24) exhibits a step at $k = 0$ that increases with $k_\perp$. This crude modelization may be cured rather simply: however, its drawbacks do not affect the results presented in this paper in any significant way.

The EI mean-field wave function as specialization of the QMC variational wave function

The QMC variational wave function, $|\Psi_{\text{QMC}}\rangle$, is the zero-gap state, $|0\rangle$, multiplied by the Jastrow factor, $J = J_1 J_2$, which accounts for one- and two-body correlations encoding the variational degrees of freedom. In this section we focus on a relevant specialization of the pair Jastrow factor, $J_2 = \prod_{i<j} \exp[u(\mathbf{r}_i, \mathbf{r}_j)]$, showing that a proper choice of the two-body term $u(\mathbf{r}, \mathbf{r}')$ allows to

recover the mean-field EI wave function to first order in u, i.e., $|\Psi_{\rm QMC}\rangle$ takes the form

$$|\Psi_{\rm EI}\rangle = \prod_k (u_k + v_k\, \hat{c}_k^\dagger \hat{v}_k)\, |0\rangle\, . \tag{55}$$

Note that the first-order restriction is consistent with the range of validity of EI mean-field theory[22]. Throughout this section we take $J_1 = 1$ and suppress spin and valley indices, as they may be included straightforwardly in the derivation, as well as we assume positive order parameter for the sake of clarity ($\eta = 0$).

To first order in the two-body factor u, the QMC wave function is

$$\Psi_{\rm QMC}(\mathbf{r}_1, \mathbf{r}_2, \ldots, \mathbf{r}_{N_{\rm e}}) = \left[1 + \sum_{i<j} u(\mathbf{r}_i, \mathbf{r}_j)\right] \Phi_0(\mathbf{r}_1, \mathbf{r}_2, \ldots, \mathbf{r}_{N_{\rm e}}), \tag{56}$$

where $N_{\rm e}$ is the number of electrons. The Slater determinant Φ_0 in real space is obtained by projecting $|0\rangle$ onto

$$\hat{\psi}^\dagger(\mathbf{r}_1)\hat{\psi}^\dagger(\mathbf{r}_2)\ldots\hat{\psi}^\dagger(\mathbf{r}_{N_{\rm e}})\, |{\rm vac}\rangle\, , \tag{57}$$

where $|{\rm vac}\rangle$ is the vacuum with no electrons present. The Fermi field annihilation operator $\hat{\psi}$ is spanned by the basis of conduction and valence band operators,

$$\hat{\psi}(\mathbf{r}) = \hat{\psi}_c(\mathbf{r}) + \hat{\psi}_v(\mathbf{r}), \tag{58}$$

with

$$\hat{\psi}_c(\mathbf{r}) = \sum_k \varphi_{ck}(\mathbf{r})\, \hat{c}_k \tag{59}$$

and

$$\hat{\psi}_v(\mathbf{r}) = \sum_k \varphi_{vk}(\mathbf{r})\, \hat{v}_k, \tag{60}$$

where the explicit effective-mass form of Bloch states φ_{ck} and φ_{vk} was given in Supplementary Note 1.

Similarly, we work out the form of $\Psi_{\rm EI}$ in real space,

$$\Psi_{\rm EI}(\mathbf{r}_1,\mathbf{r}_2,\ldots,\mathbf{r}_{N_{\rm e}}) = \left<{\rm vac}\right|\hat{\psi}(\mathbf{r}_{N_{\rm e}})\hat{\psi}(\mathbf{r}_{N_{\rm e}-1})\ldots\hat{\psi}(\mathbf{r}_1)$$
$$\times \quad \prod_k u_k\left(1+g_k\,\hat{c}^\dagger_k\hat{v}_k\right)\hat{v}^\dagger_{k_1}\hat{v}^\dagger_{k_2}\ldots\hat{v}^\dagger_{k_{N_{\rm e}}}\left|{\rm vac}\right>, \tag{61}$$

where the valence band states k_1, k_2, ..., $k_{N_{\rm e}}$, are filled up to the Dirac point in $|0\rangle$ and we defined $g_k = v_k/u_k$. To first order in g_k, $\Psi_{\rm EI}$ reads

$$\Psi_{\rm EI}(\mathbf{r}_1,\mathbf{r}_2,\ldots,\mathbf{r}_{N_{\rm e}}) = B\,\Phi_0(\mathbf{r}_1,\mathbf{r}_2,\ldots,\mathbf{r}_{N_{\rm e}})$$
$$+ \quad B\sum_k g_k\left<{\rm vac}\right|\hat{\psi}(\mathbf{r}_{N_{\rm e}})\hat{\psi}(\mathbf{r}_{N_{\rm e}-1})\ldots\hat{\psi}(\mathbf{r}_1)\;\hat{c}^\dagger_k\hat{v}_k\;\hat{v}^\dagger_{k_1}\hat{v}^\dagger_{k_2}\ldots\hat{v}^\dagger_{k_{N_{\rm e}}}\left|{\rm vac}\right>, \tag{62}$$

where $B=\prod_k u_k$ is a constant. After expanding the field operators $\hat{\psi}$ in the second row onto the basis of $\hat{v}$ and $\hat{c}$ [cf. (60) and (59)], we observe that the only non-vanishing contributions consist in products of $N_{\rm e}-1$ operators $\hat{v}$ times a single operator $\hat{c}_k$. Since $\hat{c}_k$ occurs $N_{\rm e}$ times in the $\hat{\psi}(\mathbf{r}_i)$'s, with $i=1,\ldots,N_{\rm e}$, we may write

$$\Psi_{\rm EI}(\mathbf{r}_1,\mathbf{r}_2,\ldots,\mathbf{r}_{N_{\rm e}}) = B\,\Phi_0(\mathbf{r}_1,\mathbf{r}_2,\ldots,\mathbf{r}_{N_{\rm e}}) \quad + \quad B\sum_k g_k\sum_{i=1}^{N_{\rm e}}\sum_{k'_1}\cdots\sum_{k'_{i-1}}\sum_{k'_{i+1}}\cdots\sum_{k'_{N_{\rm e}}}$$
$$\times \quad \varphi_{ck}(\mathbf{r}_i)\;\varphi_{vk'_1}(\mathbf{r}_1)\ldots\varphi_{vk'_{i-1}}(\mathbf{r}_{i-1})\varphi_{vk'_{i+1}}(\mathbf{r}_{i+1})\ldots\varphi_{vk'_{N_{\rm e}}}(\mathbf{r}_{N_{\rm e}})$$
$$\times \quad \left<{\rm vac}\right|\hat{v}_{k'_{N_{\rm e}}}\ldots\hat{v}_{k'_{i+1}}\hat{c}_k\hat{v}_{k'_{i-1}}\ldots\hat{v}_{k'_1}\;\hat{c}^\dagger_k\hat{v}_k\;\hat{v}^\dagger_{k_1}\hat{v}^\dagger_{k_2}\ldots\hat{v}^\dagger_{k_{N_{\rm e}}}\left|{\rm vac}\right>. \tag{63}$$

To make progress, we consider the generic operator identity

$$\hat{\psi}(\mathbf{r})\hat{\psi}^\dagger(\mathbf{r}')+\hat{\psi}^\dagger(\mathbf{r}')\hat{\psi}(\mathbf{r}) = \delta(\mathbf{r}-\mathbf{r}'). \tag{64}$$

Since electrons are mainly localized at honeycomb lattice sites $\mathbf{R}$ and there is—on the average—one electron per site ($N_{\rm e} = 2N$), this identity may approximately be expressed as

$$\hat{\psi}_v(\mathbf{r}_i)\hat{\psi}_v^\dagger(\mathbf{r}_j) + \hat{\psi}_v^\dagger(\mathbf{r}_j)\hat{\psi}_v(\mathbf{r}_i) \approx \frac{\delta_{\mathbf{r}_i,\mathbf{r}_j}}{aLN}, \tag{65}$$

which provides a useful representation of the identity operator $\hat{I}$ for any position of the ith electron:

$$aLN \sum_{j=1}^{N_{\rm e}} \left[\hat{\psi}_v(\mathbf{r}_i)\hat{\psi}_v^\dagger(\mathbf{r}_j) + \hat{\psi}_v^\dagger(\mathbf{r}_j)\hat{\psi}_v(\mathbf{r}_i)\right] \approx \hat{I}. \tag{66}$$

Furthermore, in the spectral representation of $\hat{I}$ we single out the contribution of momentum k,

$$\hat{I} \approx aLN \sum_{j=1}^{N_{\rm e}} \left[\varphi_{vk}(\mathbf{r}_i)\varphi_{vk}^*(\mathbf{r}_j)\left(\hat{v}_k\hat{v}_k^\dagger + \hat{v}_k^\dagger\hat{v}_k\right) + \sum_{k'\neq k}\varphi_{vk'}(\mathbf{r}_i)\varphi_{vk'}^*(\mathbf{r}_j)\left(\hat{v}_{k'}\hat{v}_{k'}^\dagger + \hat{v}_{k'}^\dagger\hat{v}_{k'}\right)\right], \tag{67}$$

which we plug into Supplementary Eq. (63). Note that, unless $\mathbf{r}_i = \mathbf{r}_j$, the contribution originating from the second addendum between square brackets of Supplementary Eq. (67) is much smaller than the one linked to the first addendum because terms that are summed over k' cancel out as they have random phases, being proportional to $\exp\left[ik'(y_i - y_j)\right]$. The outcome is

$$\begin{aligned}
\Psi_{\rm EI}(\mathbf{r}_1,\mathbf{r}_2,\ldots,\mathbf{r}_{N_{\rm e}}) \quad &= \quad B\,\Phi_0(\mathbf{r}_1,\mathbf{r}_2,\ldots,\mathbf{r}_{N_{\rm e}}) \quad + \quad B\sum_k g_k \sum_{i,j=1}^{N_{\rm e}}\sum_{k_1'}\cdots\sum_{k_{i-1}'}\sum_{k_{i+1}'}\cdots\sum_{k_{N_{\rm e}}'} \\
\times \quad & aLN\varphi_{ck}(\mathbf{r}_i)\varphi_{vk}^*(\mathbf{r}_j)\;\varphi_{vk_1'}(\mathbf{r}_1)\ldots\varphi_{vk_{i-1}'}(\mathbf{r}_{i-1})\varphi_{vk}(\mathbf{r}_i)\varphi_{vk_{i+1}'}(\mathbf{r}_{i+1})\ldots\varphi_{vk_{N_{\rm e}}'}(\mathbf{r}_{N_{\rm e}}) \\
\times \quad & \langle{\rm vac}|\,\hat{v}_{k_{N_{\rm e}}'}\ldots\hat{v}_{k_{i+1}'}\;\hat{v}_k\hat{v}_k^\dagger\hat{c}_k\;\hat{v}_{k_{i-1}'}\ldots\hat{v}_{k_1'}\;\hat{c}_k^\dagger\hat{v}_k\;\hat{v}_{k_1}^\dagger\hat{v}_{k_2}^\dagger\ldots\hat{v}_{k_{N_{\rm e}}}^\dagger\,|{\rm vac}\rangle \\
+ \quad & (\text{contact term}),
\end{aligned} \tag{68}$$

where the last contact term is negligible unless two electrons touch. Importantly, the e-h pair wave function

$$\varphi_{ck}(\mathbf{r})\varphi_{vk}^*(\mathbf{r}') = \chi_k^{cv}(\mathbf{r}-\mathbf{r}') \tag{69}$$

occurring in the second row of (68) depends on $\mathbf{r} - \mathbf{r}'$ only, which allows to decouple the sums over $\mathbf{r}_i$ and $\mathbf{r}_i - \mathbf{r}_j$, respectively. Then Supplementary Eq. (68) may be rearranged as

$$
\begin{aligned}
\Psi_{\text{EI}}(\mathbf{r}_1, \mathbf{r}_2, \ldots, \mathbf{r}_{N_\text{e}}) \quad &= \quad B\, \Phi_0(\mathbf{r}_1, \mathbf{r}_2, \ldots, \mathbf{r}_{N_\text{e}}) \quad + \quad B \sum_{\ell=1}^{N_\text{e}} aLN \sum_k g_k\, \chi_k^{cv}(\mathbf{r}_\ell) \\
\times \quad & \sum_{k'_1} \cdots \sum_{k'_{i-1}} \sum_{i=1}^{N_\text{e}} \sum_{k'_{i+1}} \cdots \sum_{k'_{N_\text{e}}} \varphi_{vk'_1}(\mathbf{r}_1) \ldots \varphi_{vk'_{i-1}}(\mathbf{r}_{i-1}) \varphi_{vk}(\mathbf{r}_i) \varphi_{vk'_{i+1}}(\mathbf{r}_{i+1}) \ldots \varphi_{vk'_{N_\text{e}}}(\mathbf{r}_{N_\text{e}}) \\
& \times \quad \langle \text{vac} |\, \hat{v}_{k'_{N_\text{e}}} \ldots \hat{v}_{k'_{i+1}}\, \hat{v}_k \hat{v}_{k'_{i-1}} \ldots \hat{v}_{k'_1}\, \hat{v}^\dagger_{k_1} \hat{v}^\dagger_{k_2} \ldots \hat{v}^\dagger_{k_{N_\text{e}}} | \text{vac} \rangle \quad + \quad (\text{contact term}),
\end{aligned} \tag{70}
$$

where, among all addenda of the mixed sum over momenta k' and index i, the only non-vanishing contributions are those permutating the annihilation operators applied to $|0\rangle$ that belong to the set $\{\hat{v}_{k_1}, \hat{v}_{k_2}, \ldots, \hat{v}_{k_{N_\text{e}}}\}$.

The final result is

$$
\Psi_{\text{EI}}(\mathbf{r}_1, \mathbf{r}_2, \ldots, \mathbf{r}_{N_\text{e}}) = B \left[1 + \sum_{\ell=1}^{N_\text{e}} \Phi_{\text{exc}}(\mathbf{r}_\ell) \right] \Phi_0(\mathbf{r}_1, \mathbf{r}_2, \ldots, \mathbf{r}_{N_\text{e}}) + (\text{contact term}), \tag{71}
$$

with the exciton wave function Φ_{exc} being defined as

$$
\Phi_{\text{exc}}(\mathbf{r}) = aLN \sum_{\text{filled } k} g_k\, \chi_k^{vc}(\mathbf{r}), \tag{72}
$$

where the sum over k is limited to those levels that are filled in $|0\rangle$ and $\mathbf{r}$ is the electron-hole distance. Supplementary Eq. (71) is a non trivial result, as it shows that the EI wave function in real space is the product of the Slater determinant Φ_0—a conventional fermionic state—times the sum over ℓ of bosonic wave functions $\Phi_{\text{exc}}(\mathbf{r}_\ell)$—the exciton wave function integrated over the whole range of possible e-h distances. The significance of Φ_{exc} relies on its Fourier transform in reciprocal space, g_k, which is the ratio of those variational factors that solve the gap equation, v_k

and u_k. The gap equation may be regarded as the many-exciton counterpart of the Bethe-Salpeter equation.

Supplementary Eq. (71) should be compared with Supplementary Eq. (56): When no pairs of electrons are in contact, QMC and mean-field EI wave functions coincide apart from a normalization factor, provided that $u(\mathbf{r}, \mathbf{r}') = 2\Phi_{\text{exc}}(\mathbf{r} - \mathbf{r}')/N$. When two electrons touch, say $\mathbf{r}_i = \mathbf{r}_j$, a discrepancy arises, which is expected since the QMC wave function enforces the cusp condition whereas the mean-field ansatz does not.

Detection of Peierls charge density wave through the order parameter ϱ_{Transl}

The QMC analysis of main text introduces the order parameter ϱ_{Transl} as a measure of the charge displacement between adjacent unitary cells along the tube axis. If the ground state is a charge density wave (CDW) with period $2a$ (the characteristic wave vector is $q = \pi/a$), then the quantum average of ϱ_{Transl} extrapolated to the thermodynamic limit is finite. In this section we discuss whether the order parameter ϱ_{Transl} may also detect a Peierls CDW with nesting vector $q = 2k_{\text{F}}$, the Fermi wave vector being located at Dirac point K.

A first issue is the commensurability of the QMC supercell with respect to the period of Peierls CDW. According to DFT calculation $k_{\text{F}} = 0.289(2\pi)/a$, hence $q = 2k_{\text{F}} = 0.422(2\pi)/a$ (folded back to first Brillouin zone) and the period is 2.37 a (Supplementary Fig. 12b). This implies that the size of the commensurate supercell exceeds our computational capability. On the other hand, the size of a smaller supercell may approximately match a multiple of the Peierls CDW

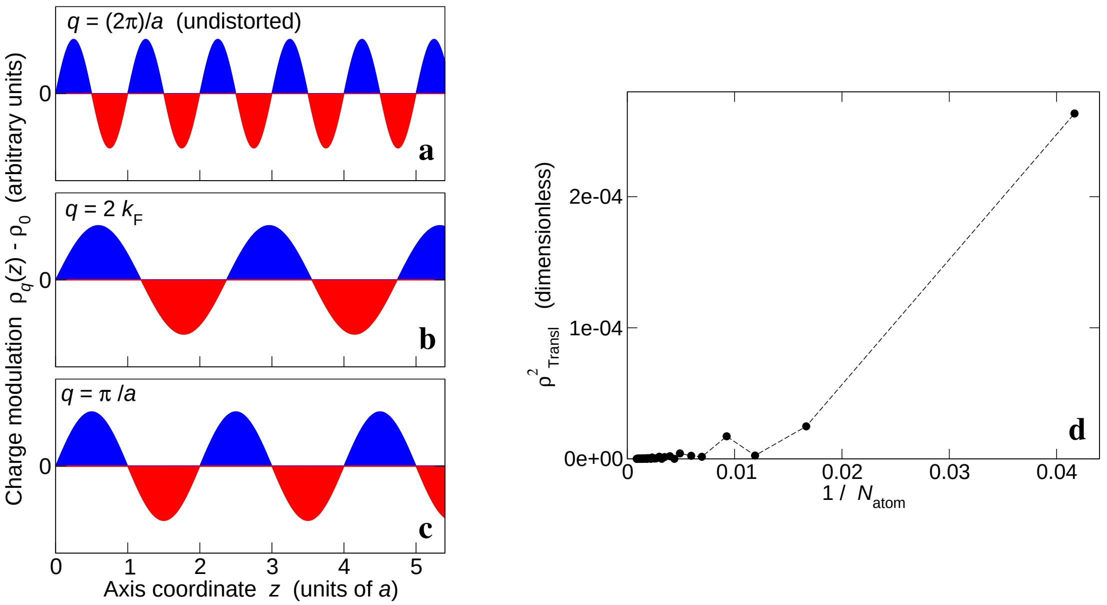

Supplementary Fig. 12 Model charge density wave ground state. **a-c** Model charge density along the axis, $n_q(z) - n_0$, vs axial coordinate, z. The density is reference from its average value, n_0, and the blue (red) colour stands for positive (negative) charge deviation. The wave vector q identifies the period of the charge density wave as $(2\pi)/|q|$: undistorted structure, $q = (2\pi)/a$ (panel a); charge density wave à la Peierls, $q = 2k_{\mathrm{F}}$, with $k_{\mathrm{F}} \equiv$ K (panel b); dimerized charge density wave, $q = \pi/a$ (panel c). **d** Square order parameter, $\left(\varrho_{\mathrm{Transl}}^{\mathrm{model}}\right)^2$, evaluated over the Peierls charge density wave model ground state, vs inverse number of atoms in the supercell, $1/N_{\mathrm{atom}}$. Dashed lines are guides to the eye.

period. This is the case e.g. of a supercell made of seven units, whose length compares with three times the period, $7.11\,a$.

The key issue is the finite-size scaling of ϱ_{Transl} averaged over the Peierls CDW ground state. To gain a better understanding, we introduce a simple model for a generic CDW. The charge density profile, $n_q(z)$, is a sinusoidal modulation of wave vector q along the axis z,

$$n_q(z) = n_{\text{mod}} \sin qz + n_0, \tag{73}$$

where n_{mod} is the modulation amplitude, n_0 is the homogeneous background, and we ignore the relaxation of the ground state occurring in a finite-size supercell. The order parameter $\varrho_{\text{Transl}}^{\text{model}}$ that fits to the model (73) is

$$\varrho_{\text{Transl}}^{\text{model}} = \frac{1}{N_{\text{cell}}} \sum_{\ell=1}^{N_{\text{cell}}} (-1)^{\ell-1} \int_{a(\ell-1)}^{a\ell} dz \, \left[n_q(z) - n_0 \right], \tag{74}$$

where N_{cell} is a number of unitary cells such that $N_{\text{cell}}a$ is approximately commensurate with the CDW period, $2\pi/q$. If N_{cell} is even, then, except for a prefactor, $\varrho_{\text{Transl}}^{\text{model}}$ is equivalent to ϱ_{Transl} as defined in the main text.

The extrapolated value of $\varrho_{\text{Transl}}^{\text{model}}$ in the thermodynamic limit, $N_{\text{cell}} \to \infty$, is trivial in two cases. For the undistorted structure, $\varrho_{\text{Transl}} = 0$ as the integral of $n_q - n_0$ over the unitary cell vanishes. This is illustrated in Supplementary Fig. 12a, where the blue (red) colour stands for positive (negative) charge deviation, $n_q(z) - n_0$. Second, for the dimerized CDW of period $2a$, which is discussed in the main text, any cell with N_{cell} even is commensurate and hence $\varrho_{\text{Transl}}^{\text{model}} = 2an_{\text{mod}}/\pi$, the integral of $n_q - n_0$ over the unitary cell exhibiting alternate sign between adjacent

cells (Supplementary Fig. 12c). In the following, in order to compare with the VQMC extrapolated order parameter ϱ_{AB} discussed in the main text, we take $n_{\text{mod}}a/\pi$ = $\varrho_{\text{AB}}/2$ = 0.00825.

We now focus on the Peierls case of nesting vector $q = 2k_{\text{F}}$ (Supplementary Fig. 12b). We assume that N_{cell} takes only those integer values closest to $(2.37)m$, with $m = 1, 2, \ldots$, which ensures that supercell and CDW periods are approximately commensurate. As illustrated by Supplementary Fig. 12d, $\left(\varrho_{\text{Transl}}^{\text{model}}\right)^2$ exhibits a complex, non-monotonic dependence on the inverse number of atoms before vanishing as $1/N_{\text{atom}} \to 0$ [here $N_{\text{atom}} = 12N_{\text{cell}}$ as the (3,3) nanotube has twelve atoms per cell]. This trend should be compared with the perfectly linear vanishing behavior exhibited by $\varrho_{\text{Transl}}^2$ in Fig. 4a of main text. We infer that, if the Peierls CDW were the actual ground state, than $\varrho_{\text{Transl}}^2$ evaluated through QMC would show some deviation from linearity, which is not observed. In conclusion, we rule out the Peierls CDW ground state.

Supplementary References

1. Ajiki, H. & Ando, T. Electronic states of carbon nanotubes. *J. Phys. Soc. Jpn.* **62**, 1255–1266 (1993).

2. Ando, T. Theory of electronic states and transport in carbon nanotubes. *J. Phys. Soc. Jpn.* **74**, 777–817 (2005).

3. Rontani, M. Anomalous magnetization of a carbon nanotube as an excitonic insulator. *Phys. Rev. B* **90**, 195415 (2014).

4. Bohnen, K., Heid, R., Liu, H. J. & Chan, C. T. Lattice dynamics and electron-phonon interaction in (3,3) carbon nanotubes. *Phys. Rev. Lett.* **93**, 245501 (2004).

5. Connétable, D., Rignanese, G., Charlier, J. & Blase, X. Room temperature Peierls distortion in small diameter nanotubes. *Phys. Rev. Lett.* **94**, 015503 (2005).

6. Ando, T. & Nakanishi, T. Impurity scattering in carbon nanotubes – Absence of backscattering. *J. Phys. Soc. Jpn.* **67**, 1704–1713 (1998).

7. Ando, T., Nakanishi, T. & Saito, R. Berry's phase and absence of backscattering in carbon nanotubes. *J. Phys. Soc. Jpn.* **67**, 2857–2862 (1998).

8. McEuen, P. L., Bockrath, M., Cobden, D. H., Yoon, Y. & Louie, S. G. Disorder, pesudospins, and backscattering in carbon nanotubes. *Phys. Rev. Lett.* **83**, 5098–5101 (1999).

9. Ando, T. Effects of valley mixing and exchange on excitons in carbon nanotubes with Aharonov-Bohm flux. *J. Phys. Soc. Jpn.* **75**, 024707 (2006).

10. Secchi, A. & Rontani, M. Wigner molecules in carbon-nanotube quantum dots. *Phys. Rev. B* **82**, 035417 (2010).

11. Abramowitz, M. & Stegun, I. A. *Handbook of mathematical functions* (Dover, New York, 1972).

12. Halperin, B. I. & Rice, T. M. The excitonic state at the semiconductor-semimetal transition. *Solid State Phys.* **21**, 115–192 (1968).

13. Ando, T. Excitons in carbon nanotubes. *J. Phys. Soc. Jpn.* **66**, 1066–1073 (1997).

14. Trevisanutto, P. E., Giorgetti, C., Reining, L., Ladisa, M. & Olevano, V. *Ab initio GW* many-body effects in graphene. *Phys. Rev. Lett.* **101**, 226405 (2008).

15. Grönqvist, J. H., Stroucken, T., Lindberg, M. & Koch, S. W. Wannier excitons signalling strong Coulomb coupling in graphene. *Eur. Phys. J. B* **85**, 395 (2012).

16. Portengen, T., Östreich, T. & Sham, L. J. Theory of electronic ferroelectricity. *Phys. Rev. B* **54**, 17452–17463 (1996).

17. Saito, R., Dresselhaus, G. & Dresselhaus, M. S. *Physical Properties of Carbon Nanotubes* (Imperial College Press, London, 1998).

18. Slonczewksi, J. C. & Weiss, P. R. Band structure of graphite. *Phys. Rev.* **109**, 272–279 (1958).

19. Suzuura, H. & Ando, T. Crossover from symplectic to orthogonal class in a two-dimensional honeycomb lattice. *Phys. Rev. Lett.* **89**, 266603 (2002).

20. Maultzsch, J. *et al.* Exciton binding energies in carbon nanotubes from two-photon photoluminescence. *Phys. Rev. B* **72**, 241402(R) (2005).

21. Spataru, C. D., Ismail-Beigi, S., Benedict, L. X. & Louie, S. G. Excitonic effects and optical spectra of single-walled carbon nanotubes. *Phys. Rev. Lett.* **92**, 077402 (2004).

22. Kohn, W. Metals and insulators. In de Witt, C. & Balian, R. (eds.) *Many-body physics*, 351–411 (Gordon and Breach, New York, 1967).

C atom label	x (Å)	y (Å)	z (Å)
1	2.101836417	0.002803388	-1.230783688
2	1.607960625	1.353495674	-1.230783688
3	1.048473311	1.821401270	0.000000000
4	-0.368220898	2.068523780	0.000000000
5	-1.053234936	1.818369640	-1.230783688
6	-1.976339544	0.715641791	-1.230783688
7	-2.102053467	-0.002752850	0.000000000
8	-1.607747246	-1.353292444	0.000000000
9	-1.048327785	-1.821179199	-1.230783688
10	0.368284950	-2.068831149	-1.230783688
11	1.053260216	-1.818547251	0.000000000
12	1.976108358	-0.715632650	0.000000000

Supplementary Table 1 Equilibrium coordinates of the twelve atoms making the unitary cell of the (3,3) carbon nanotube, after structural DFT optimization. The cell size along the tube axis, parallel to z, is 2.461566 Å.
